# Intestinal epithelial pH-sensing receptor GPR65 maintains mucosal homeostasis via regulating antimicrobial defense and restrains gut inflammation in inflammatory bowel disease

**DOI:** 10.1080/19490976.2023.2257269

**Published:** 2023-09-25

**Authors:** Gengfeng Li, Jian Lin, Xiang Gao, Huiling Su, Ritian Lin, Han Gao, Zhongsheng Feng, Huili Wu, Baisui Feng, Keqiang Zuo, Yingchuan Li, Wei Wu, Leilei Fang, Zhanju Liu

**Affiliations:** a Center for IBD Research, Shanghai Tenth People’s Hospital, School of Medicine, Tongji University, Shanghai, China; b Department of Gastroenterology, Affiliated Hospital of Putian University, Putian, China; c Department of Gastroenterology, Linfen Central Hospital of Shanxi Medical University, Linfen, China; d Department of Gastroenterology, Zhengzhou Central Hospital Affiliated to Zhengzhou University, Zhengzhou, China; e Department of Gastroenterology, The Second Affiliated Hospital of Zhengzhou University, Zhengzhou, China

**Keywords:** GPR65, intestinal epithelial cell, antimicrobial peptide, inflammatory bowel disease, antimicrobial defense

## Abstract

Intestinal epithelial cell (IEC) regulation of barrier function and mucosal homeostasis enables the establishment of a harmonious gut microenvironment. However, host-derived regulatory networks that modulate intestinal antimicrobial defenses have not been fully defined. Herein we generated mice with IEC-specific deletion of *Gpr65* (*Gpr65*^ΔIEC^) and investigated the role of epithelial GPR65 using DSS- and *C. rodentium*-induced murine colitis models. RNA sequencing analysis was conducted on colonic IECs from *Gpr65*^fl/fl^ and *Gpr65*^ΔIEC^ mice, and colonoids and colonic epithelial cell lines were used to evaluate the pH-sensing effect of GPR65. The expression of GPR65 was determined in IECs from patients with inflammatory bowel disease (IBD) and DSS colitis mice by qRT-PCR, Western blot, and immunohistochemistry, respectively. We observed that the absence of GPR65 in IECs abrogated homeostatic antimicrobial programs, including the production of antimicrobial peptides (AMPs) and defense response-associated proteins. *Gpr65*^ΔIEC^ mice displayed dysbiosis of the gut microbiota and were prone to DSS- and *C. rodentium*-induced colitis, as characterized by significantly disrupted epithelial antimicrobial responses, pathogen invasion, and increased inflammatory infiltrates in the inflamed colon. RNA sequencing analysis revealed that deletion of GPR65 in IECs provoked dramatic transcriptome changes with respect to the downregulation of immune and defense responses to bacteria. Forced AMP induction assays conducted *in vivo* or in *ex vivo* colonoids revealed that IEC-intrinsic GPR65 signaling drove antimicrobial defense. Mechanistically, GPR65 signaling promoted STAT3 phosphorylation to optimize mucosal defense responses. Epithelial cell line and colonoid assays further confirmed that epithelial GPR65 sensing pH synergized with IL-22 to facilitate antimicrobial responses. Finally, the expression of GPR65 was markedly decreased in the inflamed epithelia of IBD patients and DSS colitis mice. Our findings define an important role of epithelial GPR65 in regulating intestinal homeostasis and mucosal inflammation and point toward a potential therapeutic approach by targeting GPR65 in the treatment of IBD.

## Introduction

A symbiotic host-microbial interaction plays an essential role in the maintenance of intestinal homeostasis. The dynamic crosstalk between intestinal epithelial cells (IECs), intestinal microbiota, and local immune cells is one of the fundamental features of intestinal homeostasis. Impaired host defense against intestinal potential pathogens has been demonstrated to be associated with an increased susceptibility to inflammatory disorders such as inflammatory bowel disease (IBD).^1^ Although the precise etiology and pathophysiology of IBD are still obscure, it is well established that dysregulated intestinal barrier function and ensuing microbial invasion trigger a vicious cycle of exuberant immune responses, leading to chronic intestinal inflammation.^2^ Further insights into the molecular interactions between host and gut microbiota could potentially open new therapeutic avenues for multiple gut inflammatory diseases, such as IBD.

IECs, located at the intestinal host-microbial interface, constitute the first line of barrier against diverse environmental insults and maintain the physical segregation of microbiota from the intestinal lumen. Far more than a simple physical barrier affording metabolic and digestive functions, IECs, in conjunction with additional specialized IEC lineages, are equipped with effective mechanisms for the maintenance of intestinal homeostasis. IECs exert multiple strategies, including secretion of mucus and antimicrobial peptides (AMPs), tight junction (TJ) formation, recognition of microbial colonization, and coordination of appropriate innate and adaptive immune responses to reinforce barrier function.^3,4^ Importantly, IECs do not function in a solely intrinsic manner, but instead act as central integrators of microbial and immune signals in the intestine. Host-microbial disturbances at the intestinal epithelial interface cause multiple extraintestinal and intestinal diseases. For example, hypomorphic mutation of the autophagy gene *ATG16L1* impairs the secretory granule pathway in Paneth cells, resulting in diminished secretion of AMPs.^5^ *NOD2*, the first IBD risk allele, is required for the expression of cryptdins, and homozygous mutations in *NOD2* are highly associated with ileal Crohn’s disease (CD).^6^ However, the underlying mechanisms by which host genes regulate IEC barrier function and antimicrobial responses, limit pathogen colonization, and shape the composition of microbial communities remain poorly understood.

G protein-coupled receptors (GPCRs) represent a large family of membrane receptors that sense multitudes of extracellular signals and elicit intracellular signal transduction events, implicating in many physiological processes. A variety of metabolite-sensing GPCRs that mediate complex interactions between different dietary metabolites and intestinal immune and nonimmune cells participate in the regulation of intestinal mucosal immune and inflammatory responses.^7,8^ A recent study has demonstrated that the long-chain fatty acid receptor GPR120 promotes CD4^+^ T cell IL-10 production to inhibit intestinal inflammation.^9^ Moreover, GPR43 is found to facilitate short-chain fatty acid (SCFA)-driven Th1 cell IL-10 production,^10^ intestinal IgA response,^11^ and epithelial AMP expression,^12^ thus maintaining gut homeostasis. GPR109A, a receptor for butyrate and niacin, imposes anti-inflammatory properties in colonic macrophages and dendritic cells, and is also required for the expression of IL-18, which is responsible for epithelial integrity.^13,14^ Collectively, these studies have highlighted a crucial role of metabolite-sensing GPCRs in regulating intestinal homeostasis and provided an opportunity to initiate drug development around these overarching receptors.

Chronic intestinal inflammation is often accompanied by local acidification,^15^ which is attributed to hypoxia and increased levels of acidic metabolites resulting from anaerobic glycolysis and acidosis. An acidic microenvironment is not merely a sequelae of disease but affects the progression and resolution of inflammation.^16^ As one special subset of metabolite GPCRs, proton-sensing GPCRs consisting of GPR4, GPR65, GPR68 and GPR132 act as sensors of acidification and play an essential role in regulating physiological and pathophysiological processes.^17^ Several IBD-associated variants around *GPR65* locus have been identified in previous genome-wide association studies (GWAS),^18,19^ including one missense variant encoding an isoleucine-to-leucine substitution at codon 231 (I231L). Functional characterization of this variant further demonstrates that GPR65 I231L hampers Th17 and Th22 cell differentiation through altered cellular metabolism and augments antigen presentation in dendritic cells by influencing endo-lysosomal fusion and degradation capacity, resulting in an enhanced susceptibility to colitis.^20^ In addition, macrophages deficient in GPR65 display an impaired acidification-induced inhibition of proinflammatory cytokine production.^21^ Although GPR65 has been largely known for its function in immune cells, there is an emerging appreciation of its role in nonimmune cells, such as epithelial cells. A previous study demonstrated that *Gpr65* KO mice transferred with WT bone marrow cells exhibited more severe colitis than WT mice upon intestinal infection with the mouse pathogen *Citrobacter rodentium* (*C. rodentium*), suggesting a protective role for non-hematopoietic cell-derived GPR65 in limiting pathogen infection. Mechanistically, GPR65 I231L-harboring HeLa cells display aberrant lysosomal pH, leading to aberrant lysosomal function and impaired bacterial restriction.^22^ Altogether, these studies point out an important role of pH-sensing receptor GPR65 in the regulation of inflammatory responses and immune homeostasis. However, the exact mechanism whereby intestinal epithelial GPR65 affects the pathogenesis of IBD has yet to be fully characterized.

In the present study, we investigated the potential roles of GPR65 in regulating IEC functions and participating in intestinal homeostasis and inflammatory responses and explored its clinical relevance to the pathogenesis of human IBD. We found that GPR65 deficiency in IECs compromised intestinal antimicrobial defense, including reduced AMP production under steady state conditions. Mice with IEC-specific GPR65 deletion exhibited dysbiosis of fecal microbiota and enhanced susceptibility to both dextran sulfate sodium (DSS)- and *C. rodentium* infection-induced colitis. Furthermore, colonoids from *Gpr65*^ΔIEC^ mice exhibited a diminished capacity for defensive responses following rmIL-22 treatment *in vitro*, suggesting that IEC-intrinsic GPR65 signaling maintains intestinal antimicrobial defense. Mechanistically, GPR65 acts synergistically with IL-22 signaling to promote STAT3 phosphorylation and drive the optimal AMP response. Finally, we found a decrease in GPR65 in the inflamed epithelia of IBD patients and DSS colitis mice, highlighting that dysregulated GPR65 signaling in IECs could exacerbate the development of intestinal inflammation. Therefore, our study uncovers a novel mechanism whereby epithelial GPR65 preserves intestinal antimicrobial defense and protects against intestinal inflammation, thus providing a new therapeutic approach for the management of IBD.

## Materials and methods

### Patients and samples

All patients with CD or ulcerative colitis (UC) and healthy donors encompassed in this study were recruited from the Department of Gastroenterology, Shanghai Tenth People’s Hospital of Tongji University (Shanghai, China) from March 2021 to December 2022. The diagnosis of IBD was based on clinical symptoms, endoscopic and radiological examination, and histological findings. Crohn’s disease activity index (CDAI) and Mayo scores for UC were used to evaluate disease severity. To isolate IECs, intestinal specimens were collected from freshly resected colon tissues of IBD patients and macroscopically normal marginal colon tissues of patients undergoing therapeutic colectomy for colon cancer and other nonmalignant, non-inflammatory conditions, such as colon adenomas or multiple polyps.

This study was approved by the Institutional Review Board for Clinical Research of Shanghai Tenth People’s Hospital of Tongji University (SHSY-IEC-4.0/18–33/01). Written informed consent was obtained from all participants before the study.

### Mice

All mice were bred and maintained under specific pathogen-free conditions at the Experimental Animal Center of Tongji University School of Medicine (Shanghai, China). All mice had free access to filtered air, sterile water, and standard autoclaved food in individually ventilated cages under a 12/12 h light/dark cycle. Male and female mice between 8 and 10 weeks of age with a body weight of 20 to 25 g were used in this study. All animal experiments were performed using age- and sex-matched mice. Animal use and care were in accordance with the institutional guidelines of Tongji University. All experiments and procedures involving mice in this study were approved by the Institutional Animal Care and Use Committee of Tongji University. *Villin-*Cre mice were purchased from Shanghai Model Organisms Center, Inc. (Shanghai, China). *Gpr65*^fl/fl^ mice were generated at Shanghai Key Laboratory of Regulatory Biology (Shanghai, China) and construction details have been described in our recent study (Supplementary Figure S1a).^23^ All mice used in this study had a pure C57BL/6J genetic background.

### RNA extraction and quantitative real-time polymerase chain reaction (qRT-PCR)

Total RNA was extracted with TRIzol reagent (Invitrogen; San Diego, CA, USA), and the concentration and purity were determined to assure quality. RNA was then used as a template for reverse transcription into complementary DNA (cDNA), which was performed using a 5× All-in-one RT MasterMix Kit (Applied Biological Materials, Richmond, BC, Canada) according to the manufacturer’s instructions. RT-PCR conditions were as follows: 25°C for 10 min, 42°C for 15 min, and 85°C for 5 min. qRT-PCR was performed on the QuantStudio Dx Real-Time PCR Instrument (Applied Biosystems; Waltham, MA, USA) using a TB Green Premix Ex Taq PCR Kit (TaKaRa; Dalian, China). The cycling conditions for qRT-PCR were as follows: 30 s at 95°C, followed by 40 cycles of 5 s at 95°C, and 30 s at 60°C. The relative expression levels of target genes were normalized to the housekeeping gene *Gapdh* and calculated using the 2^–ΔΔCT^ algorithm. All primers used in this study were synthesized by Sango Biotech (Shanghai, China), and the primer sequences are detailed in Supplementary Tables 1 and 2.

### Western blot

Western blot analysis was performed as described previously.^24^ Briefly, IECs were lysed and total protein was quantified using a BCA protein assay kit (Beyotime Biotechnology; Shanghai, China). Equal amounts of protein were loaded and processed using SDS-PAGE gels (10–12%) and transferred to PVDF membranes (Millipore; Burlington, MA, USA). Membranes were then blocked in 5% nonfat milk/TBST or 5% BSA/TBST (phosphorylated protein) at room temperature for 1 h and immunoblotted with the primary antibodies overnight at 4°C. Finally, membranes were incubated with HRP-conjugated secondary antibody for 1 h at room temperature, visualized using SuperSignal West Atto chemiluminescent substrate (Pierce, Thermo Fisher Scientific; Waltham, MA, USA) and captured with the Amersham Imager 600 ECL system (GE Healthcare Life Sciences; Chicago, IL, USA). The intensities of the bands were analyzed using the ImageJ software.

Primary antibodies against REG3γ (Cat: ab198216), β-actin (Cat: sc-8432), and GPR65 (Cat: PA5–111835) were purchased from Abcam (Cambridge, UK), Santa Cruz Biotechnology (Dallas, TX, USA), and Thermo Fisher Scientific, respectively. Primary antibodies against GAPDH (Cat: 5174S), STAT3 (Cat: 4904T), phosphorylated (p)-STAT3 (Cat: 4113S), Erk1/2 (Cat: 9102S), p-Erk1/2 (Cat:4370T), mTOR (Cat: 2983T), p-mTOR (Cat: 2971S), and HRP-linked secondary antibodies against rabbit (Cat: 7074) and mouse (Cat: 7076) were purchased from Cell Signaling Technology (Danvers, MA, USA).

### Isolation of IECs

IECs were isolated from human colon specimens and murine colons as described previously.^25^ The intestines were removed and placed in ice-cold calcium- and magnesium-free PBS. The bowel tissues were then cut longitudinally, thoroughly washed in ice-cold PBS, and cut into 1.0-cm pieces. After shaking in PBS containing 5% fetal bovine serum (FBS), 5 mM EDTA (Invitrogen), and 1 mM dithiothreitol (Sigma-Aldrich; St. Louis, MO, USA) for 30 min at 37°C, the colons were shaken rigorously. The epithelial cells in the supernatant were filtered with a 100 μm strainer and spun down. The pellet of epithelial cells was layered on a 20-40% gradient Percoll-RPMI solution and centrifuged at 2000 rpm for 20 min at 20°C. IECs were collected from the interphase.

### Immunofluorescence staining

Freshly isolated intestinal specimens were fixed in 10% formalin, embedded in a paraffin block, and cut into 5 μm slices. The sections were then transferred onto glass slides for further use. After deparaffinization, rehydration, and antigen retrieval, the slides were treated with 0.3% Triton X-100 for 10 min at room temperature and blocked with 10% normal donkey serum for 1 h. Subsequently, immunofluorescence staining was performed with rabbit anti-mouse REG3γ (ab198216, Abcam) or rabbit anti-mouse lysozyme (ab108508, Abcam) overnight at 4°C. The slides were rinsed in PBS for 3 times, and were then incubated with Alexa Fluor Dye-conjugated secondary antibodies for 1 h. Finally, the sections were counterstained with DAPI and observed under a fluorescence microscope.

### 16S rRNA sequencing and data analysis

Fecal samples were collected from live mice, snap-frozen, and stored at −80°C. 16S rRNA from mouse fecal samples was extracted using an E.Z.N.A.® Soil DNA Kit (Omega Bio-Tek; Norcross, GA, USA), according to the manufacturer’s instructions. Fecal DNA quality and quantity were determined using a NanoDrop 2000 instrument (Thermo Fisher Scientific; Waltham, MA, USA). The V4–V5 variable regions of the bacterial 16S rRNA gene were amplified by PCR using the universal primer pair 515F 5′-barcode-GTGCCAGCMGCCGCGG-3′ and 907 R 5′-CCGTCAATTCMTTTRAGTTT-3′, where the barcode is an eight-base sequence unique to each sample. Purified PCR products were quantified using Qubit® 3.0 (Invitrogen) and every twenty-four amplicons with different barcodes were mixed equally. The pooled DNA product was used to construct an Illumina Pair-End library following Illumina’s genomic DNA library preparation procedures. The amplicon library was then paired-end sequenced (2 × 250) on an Illumina MiSeq platform (Shanghai Biozeron Co., Ltd.) according to standard protocols. Sequence analysis was performed using the QIIME pipeline with default settings.

### DSS-induced colitis in mice

Acute DSS-induced colitis was established in mice as described previously.^24,26^ In brief, *Gpr65*^ΔIEC^ mice and *Gpr65*^fl/fl^ littermates were administered 2% (w/v) DSS (36-50kDa, MP Biomedicals; Santa Ana, CA, USA) in drinking water for 7 days followed by 3 days of normal drinking water. Mice were monitored daily for diarrhea, weight change, and rectal bleeding. All mice were sacrificed on day 10, and the distal portions of the colon were obtained for H&E staining, immunohistochemical or immunofluorescence staining, and RNA and protein extraction.

### *C. rodentium* infection-induced colitis model in mice

*C. rodentium* (DBS100, ATCC 51459; American Type Culture Collection, Manassas, VA, USA) was grown overnight at 37°C in LB broth, harvested by centrifugation, and resuspended in PBS. *Gpr65*^ΔIEC^ mice and *Gpr65*^fl/fl^ littermates were fasted for 8 h before oral inoculation with a bacterial suspension of 2 × 10^9^/mouse *C. rodentiumC. rodentium*. Body weight was recorded daily during the period of experiment for 8 days. All mice were sacrificed on day 8. The spleen, liver, colon, and fecal pellets were collected for further analysis.

### Hematoxylin and eosin (H&E) staining and histopathological assessment

Samples of mouse intestinal tissues were collected, fixed in 10% formalin for 24 h, embedded in paraffin, and cut into 5-μm sections. After de-waxing and rehydration, the sections were stained with hematoxylin and eosin using an H&E staining kit (ab245880, Abcam). Colon sections were examined under an optical microscope (AF6000, Leica; Wetzlar, Germany), and colitis severity was evaluated under specific criteria.

For the DSS-induced colitis model, pathological scores were calculated from six parameters, with a maximum score of 12. The scoring criteria are as follows: degree of inflammation in the lamina propria of the colon (none, 0; mild, 1; moderate, 2; severe, 3), goblet cell loss (none, 0; mild/moderate, 1; severe, 2), disrupted crypts (normal, 0; hyperplastic, 1; disorganization, 2; crypt loss, 3), presence of crypt abscesses (absent, 0; present, 1), mucosal ulceration (absent, 0; present, 1), and submucosal spread to transmural involvement (none, 0; submucosal, 1; transmural, 2).^27^

For the *C. rodentium* infection-induced colitis model, pathological scores were calculated from four parameters for a maximum score of 12. The scoring criteria are as follows: epithelium change (normal, 0; mild, 1; moderate, 2; severe, 3), inflammation in the lamina propria (normal, 0; mild, 1; moderate, 2; severe, 3), area affected (none, 0; 0–25%, 1; 25–50%, 2; >50%, 3), and markers of severe inflammation (none, 0; mild submucosal inflammation or < 5 crypt abscesses, 1; mild submucosal inflammation and < 5 crypt abscesses, 2; severe submucosal inflammation or > 5 crypt abscesses or crypt branching, 2; severe submucosal inflammation and > 5 crypt abscesses or crypt branching, 3; ulceration or extensive fibrosis, 3).^28^

### Quantification of *C.* *rodentium* burden in the feces and liver

Colony-forming units (CFUs) of *C. rodentium* in the feces and liver were measured according to a previous study.^29^ Briefly, the feces or liver tissues were weighed, homogenized, and then serially diluted. CFUs from the indicated organs or feces were determined by plating on MacConkey agar plates and incubating overnight at 37°C.

### Administration of IL-17A or IL-22 *in* *vivo*

*Gpr65*^ΔIEC^ mice and *Gpr65*^fl/fl^ littermates were intraperitoneally injected with rmIL-17A (1 μg/mouse, R&D Systems; Minneapolis, MN, USA) daily or rmIL-22 (2 μg/mouse, R&D Systems) every other day for 10 days. The other two groups of mice were administered PBS as a control. Mice were sacrificed on day 10, and IECs were isolated for further experimental assessments.

### RNA sequencing and data analysis

Colonic IECs were isolated from both *Gpr65*^fl/fl^ and *Gpr65*^ΔIEC^ mice according to the above methods. Purification was performed using mouse CD45 microbeads (130-052-301, Miltenyi Biotec; Bergisch Gladbach, North Rhine-Westphalia, Germany) to eliminate contamination of leukocytes (Supplementary Figure S7a). Highly purified IECs were lysed using TRIzol, and total RNA was extracted according to the manufacturer’s instructions. The concentrations, purity, and integrity of RNA were evaluated, and sequencing libraries were constructed using the NEBNext® UltraTM RNA Library Prep Kit for Illumina® (NEB; Ipswich, MA, USA) following the manufacturer’s recommendations. Libraries were sequenced with 150-bp paired-end reads on an Illumina Novaseq platform. More details are provided in the Supplementary Methods.

### Cell lines and cell culture

Intestinal epithelial cell lines, including SW480, HT29, HCT116, HIEC, Caco2, and MC-38, were purchased from the National Collection of Authenticated Cell Cultures (Shanghai, China). These cells were cultured in 10 cm plates with DMEM supplemented with 100 U/mL penicillin/streptomycin and 10% FBS. For *in vitro* treatment with rmIL-17A or rmIL-22 (R&D Systems), cells were starved in serum-free medium for 24 h and then switched to culture medium supplemented with 5% FBS in the presence or absence of rmIL-17A (100 ng/mL) or rmIL-22 (100 ng/mL) for 12 h. The pH of the medium was adjusted with HCl or NaOH.

### Statistical analysis

Data were expressed as mean ± SEM and were analyzed using GraphPad Prism 7 (GraphPad Software; La Jolla, CA, USA). Statistical comparisons were performed using paired or unpaired 2-tailed Student *t*-test, one-way analysis of variance (ANOVA), or non-parametric Mann-Whitney *U* test. Statistical significance was defined as follows: *p < 0.05, **p < 0.01, ***p < 0.001, and ****p < 0.0001.

## Results

### Epithelial cell-specific GPR65 dictates intestinal antimicrobial programs

To study the requirements for GPR65 in regulating IEC functions, we crossed *Gpr65*^fl/fl^ mice with transgenic mice expressing intestine-specific *Villin-*Cre to generate IEC-specific *Gpr65* deficient (*Villin*^cre^*Gpr65*^fl/fl^, hereafter referred to as *Gpr65*^ΔIEC^) mice (Supplementary Figure S1a). The specific abrogation of *Gpr65* at the nucleic acid level and GPR65 protein in IECs was confirmed by nucleic acid gel electrophoresis, qRT-PCR, and flow cytometric analysis, respectively (Supplementary Figure S1b-d). *Gpr65*^ΔIEC^ mice grew normally and were indistinguishable from *Gpr65*^fl/fl^ littermates, and we did not observe overt signs of gastrointestinal abnormalities in the two groups of mice (data not shown). H&E staining of the distal ileum and colon tissues also revealed no noticeable mucosal abnormalities in unmanipulated *Gpr65*^ΔIEC^ mice compared with those in *Gpr65*^fl/fl^ mice. In addition, we found no difference in the number and morphology of goblet and Paneth cells between *Gpr65*^ΔIEC^ and control mice (Supplementary Figure S2a). Furthermore, qPCR analysis revealed comparable expression of genes encoding stem (*Lgr5*), tuft (*Dclk1*), goblet (*Muc2*), and Paneth (*Lyz1*) cell markers in colonic epithelial cells from *Gpr65*^ΔIEC^ mice compared with those from *Gpr65*^fl/fl^ mice, indicating that GPR65 did not affect the development and differentiation of specialized IEC lineages (Supplementary Figure S2b).

**Figure 1.**
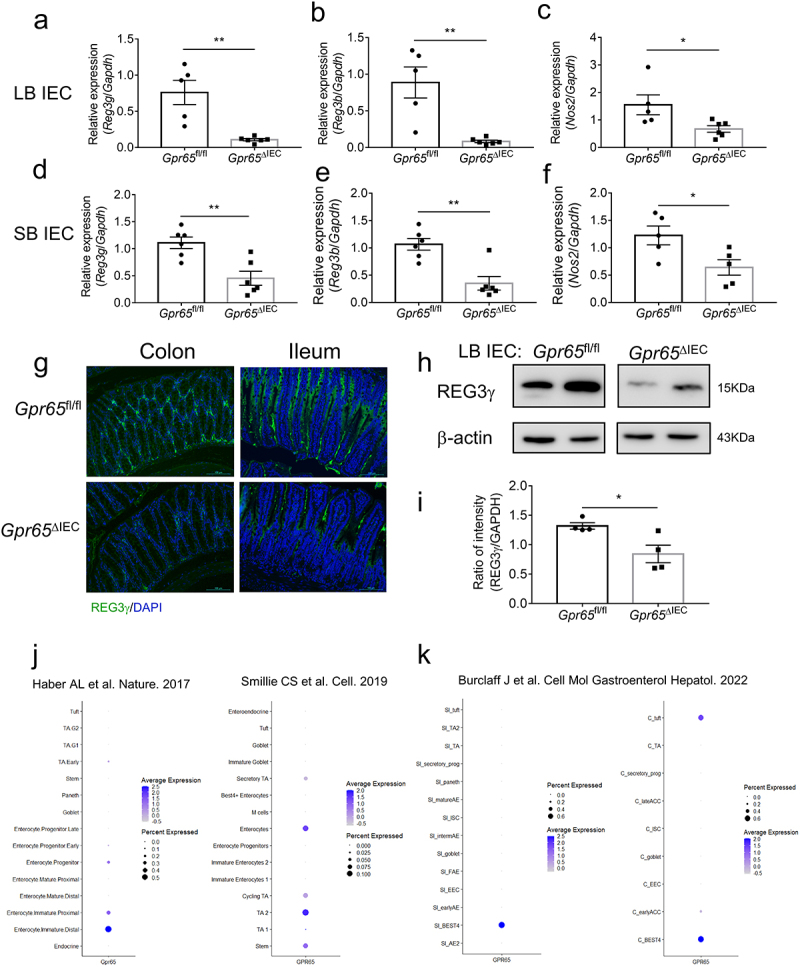
IEC-derived GPR65 preserves intestinal antimicrobial activity. (a-c) qPCR analysis of the indicated genes in IECs from the large bowels of *Gpr65*^fl/fl^ and *Gpr65*^ΔIEC^ mice. (d-f) qPCR analysis of the indicated genes in IECs from the small bowels of *Gpr65*^fl/fl^ and *Gpr65*^ΔIEC^ mice. (g) representative immunofluorescence staining of REG3γ in both large and small bowels from *Gpr65*^fl/fl^ and *Gpr65*^ΔIEC^ mice. Scale bars, 100 μm. (h, i) immunoblotting analysis and quantification of REG3γ in the IECs from the large bowels of *Gpr65*^fl/fl^ and *Gpr65*^ΔIEC^ mice. (j, k) information for cellular localization and relative expression level of GPR65 retrieved from publicly available data. *p < 0.05, **p < 0.01. Data are representative of three independent experiments.

IECs play an important role in host-microbial segregation in the gut through regulation of barrier function and acting as sentinels in intestinal homeostasis.^3^ Intriguingly, we found a significant decrease in antimicrobial genes including *Reg3g*, *Reg3b* and *Nos2* in both LB and SB IECs from *Gpr65*^ΔIEC^ mice compared with those from *Gpr65*^fl/fl^ mice (Figure 1a-f). Consistently, immunofluorescence staining of cross-sections showed markedly reduced expression of REG3γ in the epithelial layer of both ileum and colon tissues (Figure 1g). Immunoblotting further confirmed the decreased expression of REG3γ at the protein level (Figure 1h,i). However, we found no differences in the expression of defensins (*Defb1*, *Defb3*, *Defb4*) and TJ proteins (*Zo1*, *Claudin1*, *Claudin2*, *Claudin5*, *Occludin*) at the mRNA level in colonic IECs from *Gpr65*^ΔIEC^ and *Gpr65*^fl/fl^ mice (Supplementary Figure S2c). Moreover, no difference in *Il22* or *Il22bp* expression was found in colon tissues between *Gpr65*^ΔIEC^ and *Gpr65*^fl/fl^ mice, indicating that the defect in antimicrobial programs was not attributable to compromised IL-22 production (Supplementary Figure S2d).

Single-cell RNA sequencing (scRNA-seq) represents an effective method that helps to advance our understanding of human disease by comprehensively mapping risk variants to cell types and pathways.^30^ To gain deeper insight into the identity of GPR65-expressing IECs, we retrieved publicly available scRNA-seq data. These resource datasets revealed that *GPR65* is mainly expressed in enterocytes and transit-amplifying cells (Figure 1j).^30,31^ A recent study further pointed out that *GPR65* is highly expressed in BEST4-positive enterocytes^32^ which has been reported to be related to pH sensing and electrolyte balance (Figure 1k).^30^ These results thus implied that GPR65 may function as a pH-sensing receptor to regulate intestinal epithelial functions. Collectively, these data demonstrate that epithelial cell-specific GPR65 maintains intestinal antimicrobial defenses.

### GPR65 deficiency in IECs impairs the community structure of gut microbiota

**Figure 2.**
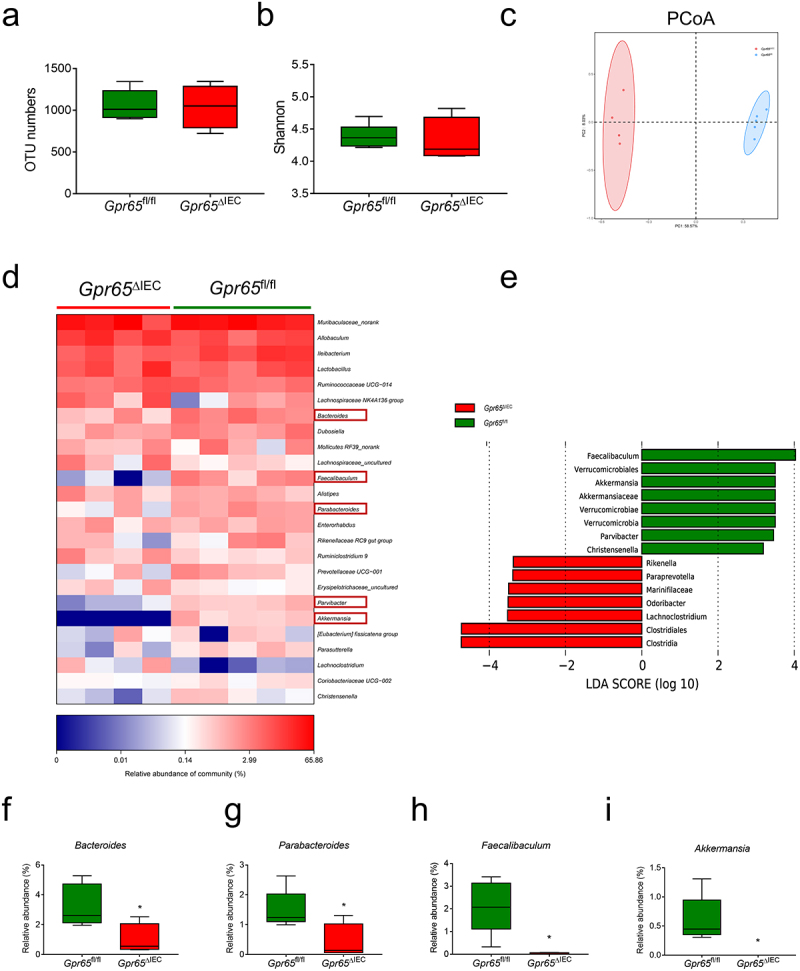
*Gpr65*^ΔIEC^ mice appear dysbiosis of fecal microbiota. (a-c) operational taxonomic unit (OTU) abundances (a), Shannon diversity index (b), and Principal coordinates analysis (PCoA) (ANOSIM analysis, *p* = 0.007) (c) of the fecal microbiota isolated from *Gpr65*^fl/fl^ (*n* = 5) and *Gpr65*^ΔIEC^ (*n* = 4) mice by 16S rRNA sequencing. Each dot represents a mouse. (d) heatmap analysis of the relative abundance of fecal microbiota at the genus level. (e) LEfSe analysis. (f-i) relative abundance of *Bacteroides*, *Parabacteroides*, *Faecalibaculum* and *Akkermansia*. *p < 0.05.

IEC-derived AMPs reinforce intestinal barrier function and are critical for the maintenance of microbiota homeostasis,^33^ and dysbiosis of gut microbiota contributes to the pathogenesis of colitis.^34^ We next performed 16S ribosomal RNA (rRNA) sequencing to characterize the fecal microbial profiles of *Gpr65*^fl/fl^ and *Gpr65*^ΔIEC^ mice. The Shannon – Wiener curve and rank-abundance distribution curve showed that the sequence depth was sufficient to capture the majority of operational taxonomic units (OTUs) and that the amount of sequencing data was sufficient to reflect microbial information in all samples (Supplementary Figure S3a,b). There was no significant difference in microbial within-community (alpha, α) diversity, as indicated by OTU numbers, Shannon index, Chao index, and Simpson index (Figure 2a,b; Supplementary Figure S3c,d). However, UniFrac distance-based principal coordination analysis (PCoA) revealed a distinct deviation between the two groups, suggesting a differential composition of the bacterial community (Figure 2c). Further analysis at the taxonomic level, including phylum and genus levels, showed a decrease in several beneficial bacteria, including *Bacteroides*, *Parabacteroides*, *Faecalibaculum* and *Akkermansia*, suggesting that GPR65 played a beneficial role in maintaining gut microbial homeostasis (Figure 2f-i; Supplementary Figure S3e). Taken together, these data indicate that depletion of GPR65 in IECs affects the community structure of gut microbiota and causes microbiota dysbiosis, and that the latter may be due to the disrupted production of AMPs in these mice.

### Ablation of epithelial GPR65 predisposes mice to DSS-induced acute colitis but protects against AOM/DSS-induced CAC

**Figure 3.**
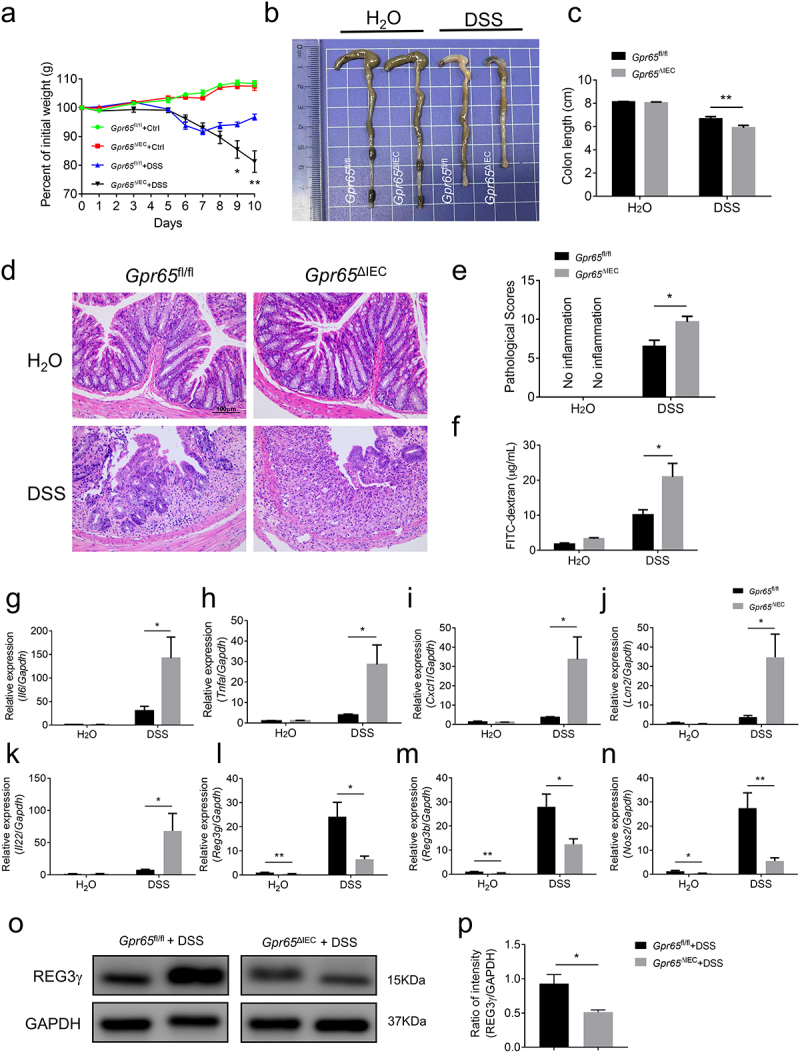
Mice deficient for epithelial GPR65 are prone to DSS-induced colitis. *Gpr65*^ΔIEC^ mice and *Gpr65*^fl/fl^ littermates (*n* = 6 in each group) were administered 2% DSS in drinking water for 7 days, followed by 3 days of regular water. (a) the body weight changes of *Gpr65*^fl/fl^ and *Gpr65*^ΔIEC^ mice during DSS-induced colitis. (b) gross morphology of colons on day 10 when mice were sacrificed. (c) colon length on day 10 of the colitis model. (d) representative sections of the distal colon tissues after H&E staining. Scale bars, 100 μm. (e) pathological scores of the colon sections were calculated as indicated. (f) FITC-dextran (μg/mL) in sera. (g-k) relative *Il6*, *Tnfa*, *Cxcl1*, *Lcn2* and *Il22* mRNA expression in the distal colon tissues of indicated groups of mice. (l-n) relative *Reg3g*, *Reg3b* and *Nos2* mRNA expression in the colonic IECs of indicated groups of mice. (o-p) immunoblotting analysis and quantification of REG3γ in the colonic IECs. *p < 0.05, **p < 0.01. Data are representative of three independent experiments.

Given that epithelial cell-derived GPR65 is associated with intestinal antimicrobial defense, we sought to determine its role in the development of colitis. Towards this end, *Gpr65*^ΔIEC^ mice and *Gpr65*^fl/fl^ littermates were subjected to 2% DSS in drinking water to induce acute colitis. In contrast to *Gpr65*^fl/fl^ counterparts, *Gpr65*^ΔIEC^ mice displayed more pronounced weight loss, colon shortening, and histo-architectural alterations, characterized by increased inflammatory cell infiltrates, crypt loss, edema, and extensive ulceration (Figure 3a-e). Consistent with our histological findings showing impaired intestinal barrier function, we observed a significant increase in serum levels of FITC-dextran in *Gpr65*^ΔIEC^ mice compared to controls (Figure 3f). As expected, the mRNA levels of proinflammatory mediators including *Il6*, *Tnfa*, *Cxcl1*, *Lcn2* and *Il22* were markedly elevated in the colon tissues of *Gpr65*^ΔIEC^ mice (Figure 3g-k). Moreover, infiltration of inflammatory cells, such as CD4^+^ T cells, MPO^+^ neutrophils, and F4/80^+^ macrophages, was significantly increased in the inflamed colon of *Gpr65*^ΔIEC^ mice following DSS treatment, suggesting enhanced inflammatory responses in the colon tissues (Supplementary Figure S4a,b). In contrast, colonic IEC initiation of antibacterial responses upon DSS insult, such as production of AMPs, was grossly disrupted in *Gpr65*^ΔIEC^ mice compared to that in *Gpr65*^fl/fl^ mice (Figure 3l-p). These data indicate that the selective absence of GPR65 in IECs leads to increased susceptibility to damage-induced inflammation.

Perpetuation of intestinal inflammation is associated with cancer development.^35^ We further evaluated the role of epithelium-derived GPR65 in chronic intestinal tumorigenesis by employing an AOM/DSS-induced CAC model. Unexpectedly, we observed a distinct phenotype of *Gpr65*^ΔIEC^ mice in CAC tumorigenesis, relative to the acute colitis model. As shown in Supplementary Figure S5a-f, *Gpr65*^ΔIEC^ mice displayed less weight loss and relieved gross colonic pathologies, as reflected by reduced tumor loads and decreased size of adenomas as opposed to *Gpr65*^fl/fl^ littermates. Histopathological analysis by H&E staining showed significant mitigation of atypical hyperplasia and adenocarcinoma formation in AOM/DSS-treated *Gpr65*^ΔIEC^ mice. Moreover, loss of epithelial GPR65 resulted in decreased expression of proliferative markers, including Ki67 and PCNA, in adenoma-bearing colon tissues (Supplementary Figure S5g). These results suggest a detrimental role for epithelium-derived GPR65 in chronic colitis-associated tumorigenesis.

Taken together, these data reveal the dichotomous functions of epithelial GPR65 during acute intestinal inflammation and chronic intestinal inflammation-induced tumorigenesis, indicating a context-dependent role of epithelial GPR65 in different pathogenic milieu.

### GPR65 deficiency in IECs renders mice susceptible to *C. rodentium*-induced colitis

**Figure 4.**
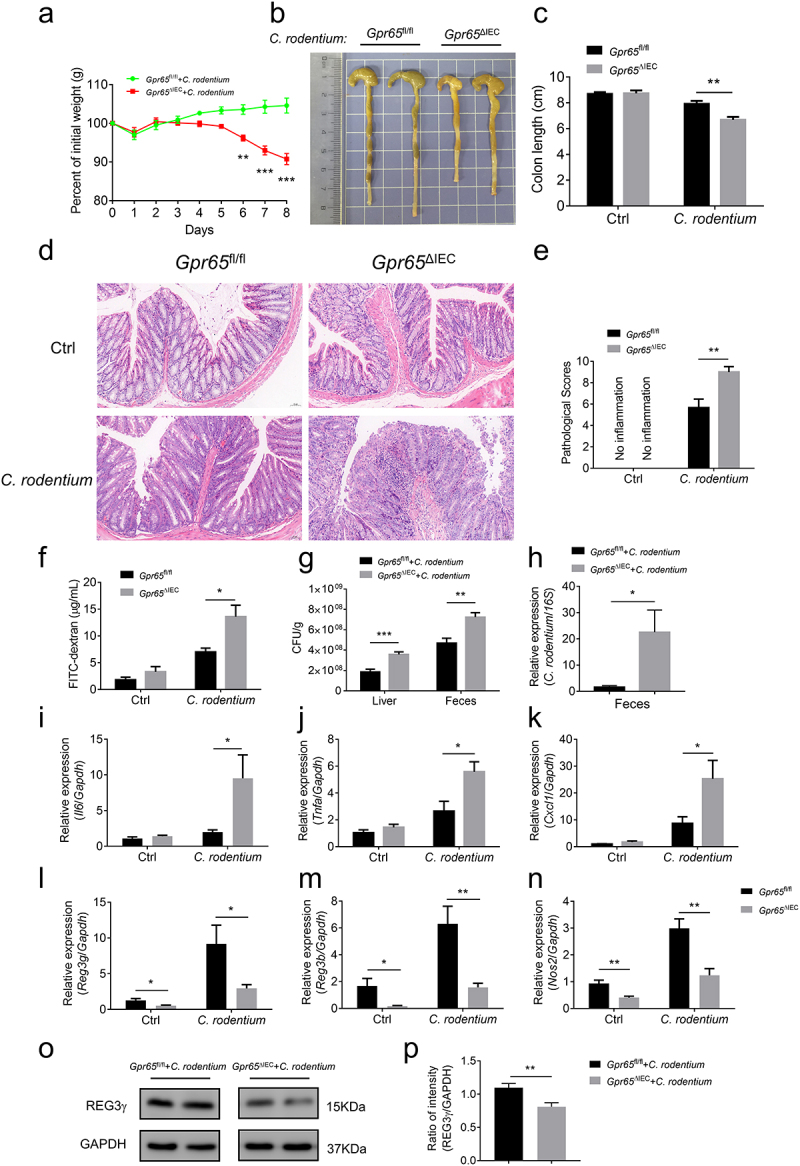
Mice lacking epithelial GPR65 are vulnerable to *C. rodentium*-induced colitis. *Gpr65*^ΔIEC^ mice and *Gpr65*^fl/fl^ littermates (*n* = 6 in each group) were orally infected with *C. rodentium* (2 × 10^9^ CFU/mouse) after fasting for 8 h, and sacrificed at day 8. (a) the body weights of mice were monitored daily. (b, c) gross morphology and colon length in the indicated groups of mice. (d) representative images of the distal colon tissues after H&E staining. Scale bars, 50 μm. (e) pathological scores of the colon sections were calculated as indicated. (f) FITC-dextran (μg/mL) in sera. (g) *C. rodentium* (Cfu/g) in the liver and feces were measured. (h) qPCR analysis of *C. rodentium* expression relative to *16S* rRNA in the feces. It should be noted here that qPCR measures relative expression between samples, not the actual bacterial numbers or CFUs. (i-k) relative *Il6*, *Tnfa* and *Cxcl1* mRNA expression in the distal colon tissues of indicated mice. (l-n) relative *Reg3g*, *Reg3b* and *Nos2* mRNA expression in the colonic IECs of indicated groups of mice. (o-p) immunoblotting analysis and quantification of REG3γ in the colonic IECs. *p < 0.05, **p < 0.01, ***p < 0.001. Data are representative of three independent experiments.

*C. rodentium* is a noninvasive, attaching and effacing (A/E) enteric pathogen in mice, which mirrors two human pathogens, namely enteropathogenic *Escherichia coli* (*E. coli*) (EPEC) and enterohemorrhagic *E. coli* (EHEC). *C. rodentium* predominantly infects the distal large intestine of mice and usually causes dysbiosis and mucosal inflammation which resembles IBD in many aspects.^36^ Previous study has confirmed that IL-22-mediated IEC production of REG3γ and REG3β provides early phase of host defense against *C. rodentium*.^37^ Therefore, we questioned whether GPR65 plays an essential role in mucosal immunity against enteric bacteria. To this end, we leveraged *C. rodentium* infection-induced colitis model. As expected, *Gpr65*^ΔIEC^ mice suffered more weight loss, colon length shortening, and apparent splenomegaly compared with control littermates (Figure 4a-c; Supplementary Figure S6a). H&E staining of the distal colon sections from *Gpr65*^ΔIEC^ mice displayed higher pathological scores, as evidenced by intensive inflammation, ulceration, and epithelial hyperplasia compared with *Gpr65*^fl/fl^ controls (Figure 4d,e). Furthermore, *Gpr65*^ΔIEC^ mice showed an increased number of infiltrating leukocytes in the colon tissues, significantly higher levels of FITC-dextran in the sera, and drastically augmented colonization of *C. rodentium* in both the liver and feces 8 d post-infection (Supplementary Figure S6b,c; Figure 4f-h). Consistently, proinflammatory cytokines and chemokines consisting of *Il6*, *Tnfa*, *Il22* and *Cxcl1* were markedly elevated in *Gpr65*^ΔIEC^ mice, whereas IEC production of AMPs was significantly perturbed after DSS exposure (Figure 4i-p; Supplementary Figure S6d). Collectively, these data confirm a protective effect of epithelial GPR65 on intestinal mucosal inflammation in a bacterium-driven colitis model.

**Figure 5.**
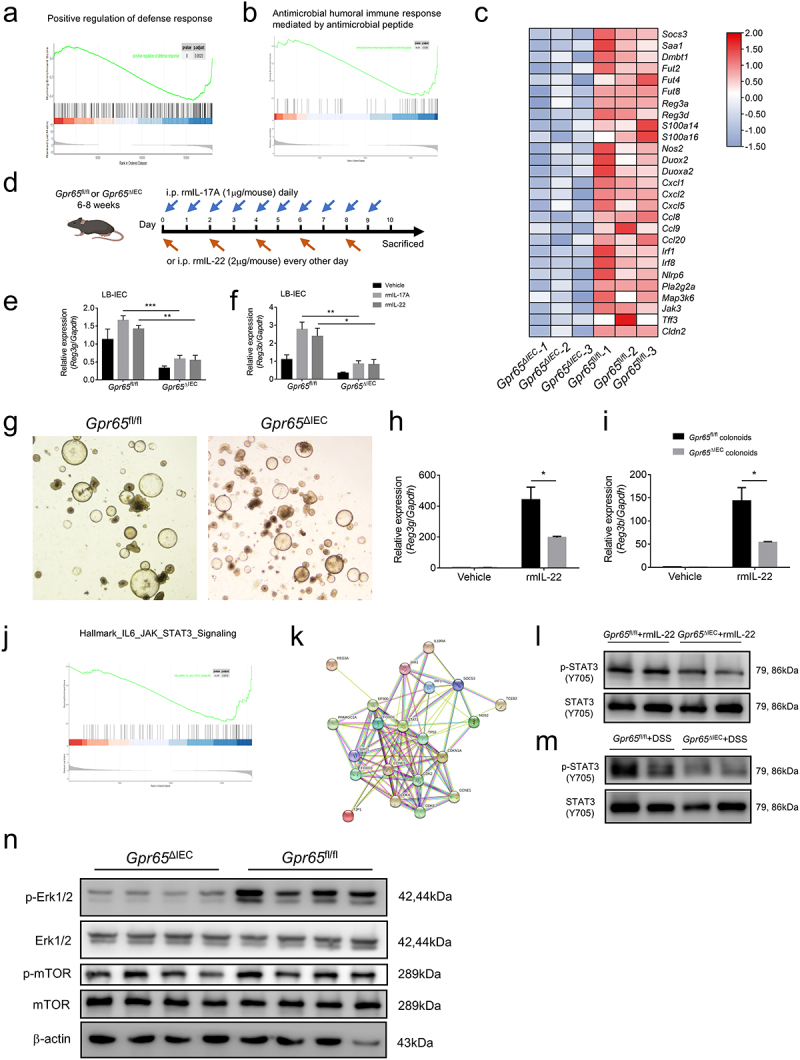
GPR65 deficiency drives distinct IEC transcriptional programs and compromises the downstream STAT3 signaling. colonic IECs were isolated from *Gpr65*^ΔIEC^ mice and *Gpr65*^fl/fl^ littermates for RNA sequencing analysis. (a, b) GSEA analysis. (c) Heatmap of DEGs related to IEC antimicrobial gene profiles. (d) a schematic overview of the AMP induction assay by systemic injection with rmIL-17A or rmIL-22. (e, f) qPCR analysis of relative mRNA expression of *Reg3g* and *Reg3b*. (g) representative microscopical photographs of colonoids from *Gpr65*^ΔIEC^ and littermate *Gpr65*^fl/fl^ mice on day 10 of culture. Original magnification: × 40. (h, i) the colonoids from *Gpr65*^ΔIEC^ and *Gpr65*^fl/fl^ mice were stimulated with or without rmIL-22 (100 ng/mL) for 24 h. The relative mRNA expression of *Reg3g* and *Reg3b* was detected by qPCR analysis. (j) GSEA analysis for Hallmark_JAK_STAT3_Signaling. (k) STAT3-focused interaction network of genes downregulated by GPR65 signaling. (l) colonic IECs were isolated from *Gpr65*^ΔIEC^ mice and *Gpr65*^fl/fl^ littermates, and stimulated with rmIL-22 (100 ng/mL) for 15 min. The protein levels of p-STAT3 and STAT3 were determined by immunoblotting analysis. (m) immunoblotting analysis of p-STAT3 and STAT3 in the colonic IECs from the indicated groups of mice. (n) immunoblotting analysis of p-Erk1/2, Erk1/2, p-mTOR, mTOR, and β-actin in the colonic IECs from the indicated groups of mice. *p < 0.05, **p < 0.01, ***p < 0.001. Data are representative of three independent experiments.

To decipher the molecular basis that mediates the effects of GPR65 on IEC physiology as well as colitis and chronic CAC development, we performed RNA sequencing on colonic epithelial samples from *Gpr65*^ΔIEC^ and *Gpr65*^fl/fl^ mice after magnetic cell sorting to exclude the contamination of CD45^+^ leukocytes (Supplementary Figure S7a). Principal component analysis (PCA) clustering analysis, heatmap, and volcano plot of differentially expressed genes (DEGs) revealed distinct genome-wide expression profiles between the two groups of mice (Supplementary Figure S7b-d). Notably, the pathway enrichment analysis of downregulated transcripts in *Gpr65*^ΔIEC^ mice implied that the enriched processes with high significance were related to the response to interferon-gamma, inflammatory response, and positive regulation of immune response (Supplementary Figure S7e). We also conducted gene set enrichment analysis (GSEA) for Gene Ontology (GO) biological processes and Kyoto Encyclopedia of Genes and Genomes (KEGG) pathways to interpret the functional relevance of transcriptome changes. In the gene list ranked by gene expression fold changes, we found that GO biological processes related to positive regulation of defense response and antimicrobial humoral immune response mediated by antimicrobial peptides were significantly downregulated in IECs from *Gpr65*^ΔIEC^ mice (Figure 5a,b). Furthermore, predefined GO and KEGG gene sets for positive regulation of immune response, defense response to bacterium, and inflammatory bowel disease were also drastically downregulated (Supplementary Figure S8a-c). Further detailed analysis of the transcripts revealed that the core enrichment genes comprised a prominent signature of IL-22-induced genes, including AMPs, CXC chemokines, and members of the calcium-binding S100 protein family that encodes calprotectin (S100a8 and S100a9), suggesting strikingly impaired epithelial innate defense in *Gpr65*^ΔIEC^ mice (Figure 5c). Notably, several canonical IL-22 responsive genes such as *Reg3b*, *Reg3g*, *Nos2*, *Fut2* and *Socs3* were markedly downregulated, consistent with our above findings, as shown in Figure 1.

#### GPR65 deficiency compromises the downstream STAT3 signaling in IECs

To investigate whether the stark impairment of antimicrobial defense was due to the intrinsic deficiency of GPR65 in IECs, we performed an AMP induction assay by intraperitoneal injection of rmIL-17A or rmIL-22 (Figure 5d). As shown in Figure 5e,f, forced induction of antimicrobial defensive responses, such as production of AMPs, was substantially perturbed in IECs from *Gpr65*^ΔIEC^ mice, in contrast to *Gpr65*^fl/fl^ littermates. We further generated colonic organoids (colonoids) from both *Gpr65*^ΔIEC^ and *Gpr65*^fl/fl^ mice and found no morphological differences, suggesting normal proliferation, development, and differentiation of IECs in *Gpr65*^ΔIEC^ mice (Figure 5g). Consistently, IL-22-mediated induction of AMP production in *Gpr65*^fl/fl^ colonoids remained intact but was markedly disrupted in *Gpr65*^ΔIEC^ colonoids (Figure 5h,i). Therefore, *in vivo* induction assays and *ex vivo* organoid data highlight that the impaired antimicrobial defense in *Gpr65*^ΔIEC^ mice can be attributed to the loss of IEC-intrinsic GPR65 signaling.

To gain further insight into the signaling pathways underlying the molecular mechanisms, we conducted GSEA and protein-protein interaction (PPI) network analyses. Predefined Hallmark and KEGG gene sets for the JAK-STAT signaling pathway were significantly downregulated in IECs from *Gpr65*^ΔIEC^ mice (Figure 5j; Supplementary Figure S8d). PPI network analysis further identified that STAT3, which was at the central site in the interaction network, was an impaired upstream essential node protein (Figure 5k). Colonic IECs isolated from *Gpr65*^ΔIEC^ mice exhibited lower levels of STAT3 phosphorylation than *Gpr65*^fl/fl^ controls following direct IL-22 stimulation. Likewise, upregulation of p-STAT3 was significantly impaired in colonic IECs from *Gpr65*^ΔIEC^ mice after induction of DSS colitis (Figure 5l,m). Numerous studies have demonstrated the crucial role of STAT3-mediated downstream signaling in response to IL-22 in participating in regulation of IEC AMP expression and mucosal wound healing.^37^ These data suggest that GPR65 functions in an IL-22-STAT3-dependent manner. We also defined associated pathways and found a decreased phosphorylation level of Erk1/2 in *Gpr65*^ΔIEC^ IECs (Figure 5n). Taken together, these in silico analyses, combined with wet experiments, indicate that GPR65 promotes IEC-mediated antimicrobial defense through the JAK-STAT3 signaling pathway.

#### pH sensing role of epithelial GPR65 regulates AMP production *in vitro*

**Figure 6.**
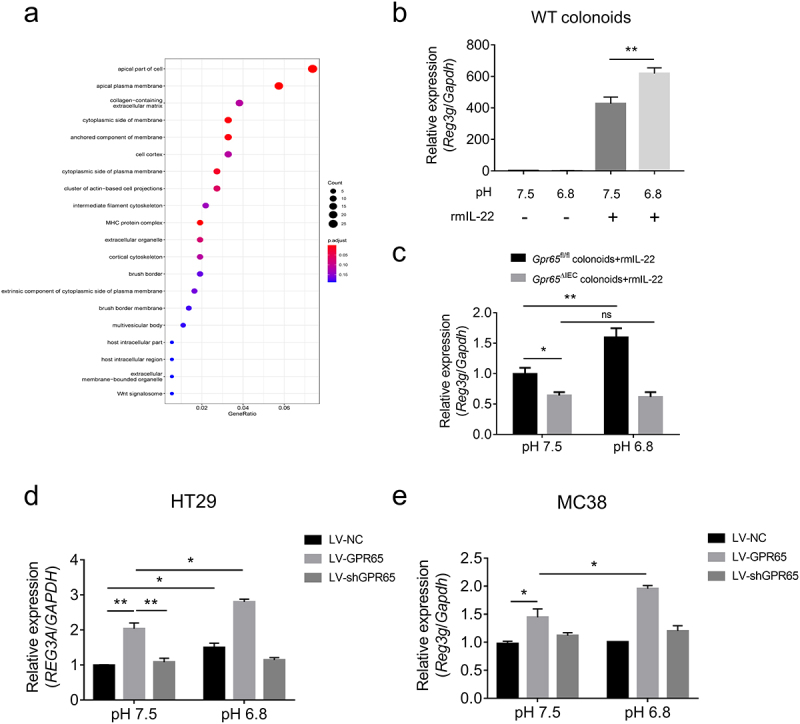
pH sensing role of epithelial GPR65 in regulating AMP expression *in vitro*. (a) GO cellular component analysis for DEGs. (b) qPCR analysis of relative *Reg3g* in WT colonoids under the indicated conditions. (c) colonoids isolated from *Gpr65*^ΔIEC^ and *Gpr65*^fl/fl^ mice were stimulated with rmIL-22 under the neutral or acidic conditions, and *Reg3g* mRNA was examined by qPCR analysis. (d, e) HT29 and MC38 cells were transfected with lentivirus expressing GPR65 shRNA (LV-shGPR65), GPR65 (LV-GPR65) and negative control (LV-NC), respectively, and were stimulated with rmIL-22 under the neutral or acidic conditions. The relative mRNA expression of *REG3A* or *Reg3g* was examined by qPCR. *p < 0.05, **p < 0.01, ***p < 0.001, ****p < 0.0001. ns, not significant. Data are representative of three independent experiments.

We reanalyzed the RNA sequencing data using GO cellular component analysis and found that the majority of differential transcripts in IECs encode proteins that were localized to the apical part of the cell or apical plasma membrane (Figure 6a), suggesting that the major action site of GPR65 was localized in the cell membrane. We then conducted *in vitro* AMP induction assays in *ex vivo* cultured colonoids as well as gut epithelial cell lines to determine the pH-sensing role of GPR65 in the regulation of IEC antimicrobial defense. To date, the specific physiological ligand of GPR65 is yet to be determined, and protons appear to be the main ligands. As shown in Figure 6b, a weak acidic stimulus did not evoke *Reg3g* expression in the absence of rmIL-22 in WT colonoids, whereas combination treatment led to enhanced *Reg3g* in contrast to IL-22 alone. However, induction of *Reg3g* was markedly disrupted in colonoids from *Gpr65*^ΔIEC^ mice, even under acidic conditions of pH 6.8 (Figure 6c). The epithelial cell lines HT29 and MC38 were also confirmed to upregulate *REG3A* and *Reg3g* expression in response to IL-22, respectively (Supplementary Figure S8e,f). Likewise, we found that, in the presence of IL-22, lentivirus (LV)-mediated overexpression of GPR65 upregulated the expression levels of *REG3A* and *Reg3g* in both HT29 and MC38 cell lines under neutral conditions, and the increased levels were much higher upon an acidic pH shift. Overall, these results suggest that GPR65-mediated pH sensing plays an essential role in promoting antimicrobial defenses in IECs.

#### GPR65 is decreased in inflamed epithelia of IBD patients and DSS-induced colitis mice

**Figure 7.**
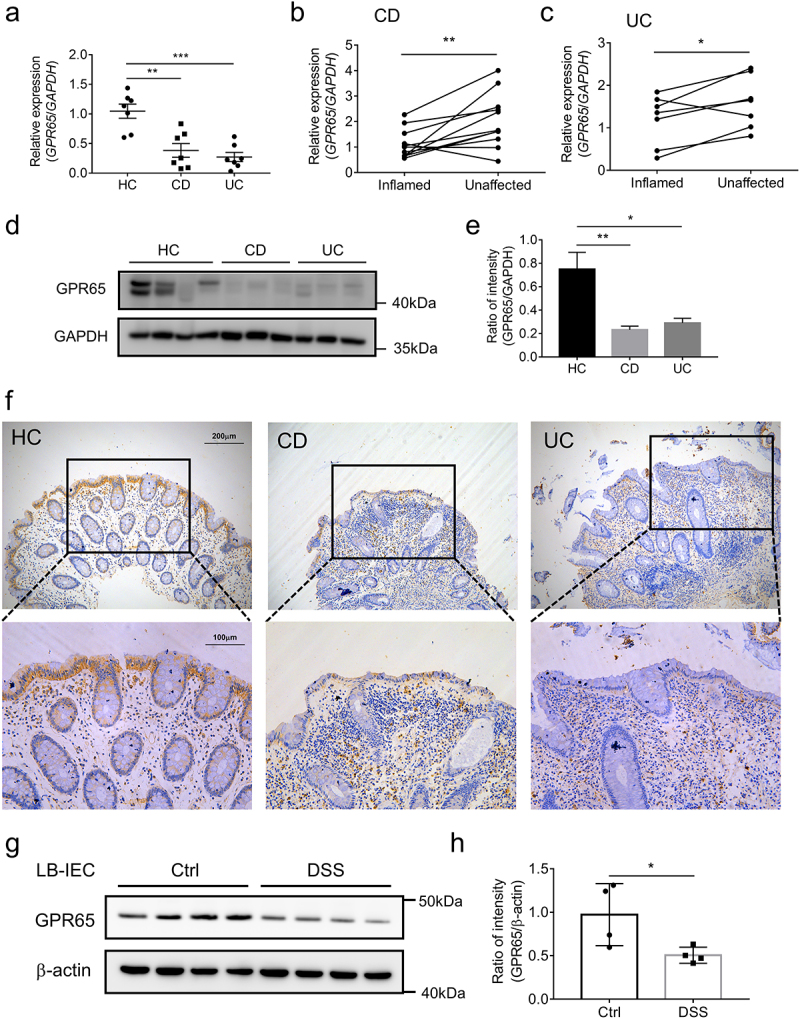
GPR65 is decreased in inflamed mucosa from patients with active IBD and DSS-induced colitis mice. colonic IECs were isolated to extract total RNA and protein. Expression levels of GPR65 were determined by qRT-PCR and Western blot, respectively. (a) relative mRNA expression level of *GPR65* in freshly isolated IECs from inflamed sites of patients with CD and UC and relatively normal sites of patients with colon cancer as controls. (b, c) relative mRNA expression levels of *GPR65* in paired inflamed and non-affected IECs from patients with CD and UC. (d, e) immunoblotting analysis of GPR65 in inflamed IECs from CD and UC patients and relatively normal IECs from patients with colon cancer. (f) representative sections were obtained from normal colonic mucosa of healthy controls (the left panel) and inflamed colonic mucosa of patients with active CD (the middle panel) or active UC (the right panel), and then stained for GPR65 by immunohistochemistry. Scale bars, 200 μm (inset, 100 μm). (g, h) colonic IECs were isolated from DSS-induced colitis mice and control mice, respectively, and the protein levels of GPR65 were determined by Western blot with β-actin as a reference. *p < 0.05, **p < 0.01, ***p < 0.001. Data are representative of three independent experiments.

Finally, we sought to address the clinical relevance to human IBD. To this end, IECs were isolated from freshly resected colon tissues of IBD patients and macroscopically normal marginal colon tissues of patients undergoing therapeutic colectomy for colon cancer and other nonmalignant, non-inflammatory conditions, such as colon adenomas or multiple polyps. We found a marked decrease in GPR65 mRNA transcripts in inflamed colonic epithelia in both CD and UC patients compared to that in normal controls (Figure 7a). Likewise, a consistent trend of reduced GPR65 mRNA levels was also observed in inflamed versus unaffected IECs from patient-matched samples (Figure 7b,c). At the protein level, we found that freshly isolated IECs from inflamed colon tissues of IBD patients displayed a dramatic decrease in GPR65 expression compared to those from non-inflamed normal controls (Figure 7d,e). Furthermore, immunohistochemical staining confirmed the decreased expression of GPR65 in inflamed epithelia from IBD patients, albeit more GPR65^+^ leukocytes present in the lamina propria, which was consistent with our previous study,^23^ suggesting a distinct role for GPR65 in regulating epithelial and immune cell functions (Figure 7f). We then investigated the association between GPR65 expression and colitis in mouse models. For this purpose, we isolated colonic epithelial cells from both DSS-induced colitis mice and control mice and found that the protein levels of GPR65 were markedly downregulated in IECs from colitis mice (Figure 7g,h). These mouse results aligned with human data, suggesting decreased expression of epithelial GPR65 during intestinal mucosal inflammation. Therefore, according to the preceding results, it is reasonable to postulate that dysregulation of the proton-sensing pathway in human IECs may account for some aspects of increased susceptibility to IBD.

### Discussion

The gastrointestinal tract acts as a huge reservoir for diverse communities of microorganisms, and fine-tuned regulation of antimicrobial defenses is required to maintain a healthy and balanced gut ecosystem. However, there is a gap in the knowledge on how IECs integrate different signals from intestinal circumstances to establish immune tolerance to innocuous commensals while mounting appropriate immune responses against pathogen invasion. In the current study, we demonstrated that GPR65 sensation of pH signals from the gut microenvironment preserves the antimicrobial responses in IECs. The absence of GPR65 in IECs causes a diminishment in antimicrobial defense and subsequent dysbiosis, which is responsible for the disrupted barrier integrity and enhanced susceptibility to colitis.

The intestinal epithelium is faced with a continuous and complex microbial challenge, and IECs cope with this challenge in part by producing mucus and a diverse arsenal of AMPs that promote physical separation of commensal bacteria and the host in the intestine.^33,38^ The functional relevance of GPR65 has been well dissected in immune cells, whereas whether IECs rely on GPR65 signaling to exert immune defensive functions remains elusive. Herein we generated mice with GPR65-specific deletion in IECs and carefully examined the compositions of diverse specialized IEC lineages, apparently being no difference. Intriguingly, we found significantly decreased AMP expression in IECs of *Gpr65*^ΔIEC^ mice. Immunofluorescence staining of REG3γ showed that GPR65 mainly disturbed the expression of REG3γ in the middle-bottom crypt. Previous studies have demonstrated that enterocytes at villus bottoms, which are not terminally differentiated cells, strongly express an antibacterial gene program.^39^ These results also indirectly corroborated the in silico analysis of previously uploaded datasets, demonstrating that GPR65 is mainly expressed in absorptive enterocytes and transit-amplifying cells.^30,31^ It is noteworthy that although additional specialized IEC lineages including Paneth cells and goblet cells are recognized as pivotal AMP-producing effector cells, absorptive enterocytes that represent the majority of IECs in the epithelial layer are important sources of AMPs other than exerting metabolic and digestive function.^3,40^ Therefore, we propose that the impaired antimicrobial defense in *Gpr65*^ΔIEC^ mice may be attributed to the overall disruption of IEC antimicrobial programs, but not derived from the alteration of specific secretory epithelial lineages. Additionally, the forced AMP induction assay conducted *in vivo* or in *ex vivo* colonoids further substantiated that IEC-intrinsic GPR65 signaling orchestrated intestinal AMP expression.

Impaired intestinal innate defense, concurrent with dysbiotic microbiota, usually results in elevated susceptibility to colitis. As expected, selective depletion of GPR65 in IECs predisposes mice to colitis triggered by DSS and *C. rodentium* infection. IEC initiation of antimicrobial responses upon inflammatory insults was much weaker in *Gpr65*^ΔIEC^ mice than in *Gpr65*^fl/fl^ controls. Impaired colonization resistance against *C. rodentium* also indicated that the presence of epithelial GPR65 is central for anatomical containment and prevention of pathogen dissemination. These *in vivo* studies verified the important role of epithelial GPR65 in the regulation of intestinal homeostasis and inflammation. A recent study revealed high similarities between *C. rodentium*-induced colitis model and human IBD, and accentuated the important role of IL-22-driven defensive response in the amelioration of epithelial dysfunction.^41^ Thus, we suppose that the disrupted function of GPR65 in human IECs may also increase a risk of intestinal inflammation.

It is commonly accepted that expression of AMPs is dependent on IEC-intrinsic pattern-recognition receptor (PRR) signaling as well as cytokine signals from innate lymphoid cells (ILCs) and T helper type 17 (Th17) cells in the underlying lamina propria.^42^ Loss of epithelial Myd88, an adaptor protein of TLRs signaling, compromises REG3γ expression and results in increased bacterial translocation.^43–45^ Besides, accumulating lines of evidence have demonstrated that IL-22 produced by ILC3 and Th17 cells promotes IEC expression of AMPs.^37,40,46,47^ However, there are controversial views regarding the signal origins in regulation of epithelial AMP expression, with other studies illustrating that TLR responses occurring in myeloid cells rather than IECs are responsible for AMP expression.^48^ Moreover, another study reported that AMP gene programs in colonic IECs are induced by inflammatory cytokines (IL-22 and IFN-γ) rather than TLR signaling.^49^ Thus, it is still not yet clear why regulation of AMP expression in IECs entails a complicated and tightly regulatory network.^42^ Our previous study found that the SCFAs-GPR43 signaling could regulates IEC expression of AMP and intestinal homeostasis.^12^ Recent published work also addresses a protective role of IEC-intrinsic IL-1R in synergy with IL-22R signaling to enhance AMP expression.^50^ Here, RNA sequencing analysis further confirmed a defect of antimicrobial programs in IECs from *Gpr65*^ΔIEC^ mice and pinpointed the impaired STAT3 signaling accordingly. STAT3 was found to link IL-22 signaling in IECs, which mediates AMP expression and mucosal wound healing.^40,51^ Our *in vitro* experiments indicated that acidosis-activated GPR65 signaling alone does not induce AMP expression, but rather synergizes with IL-22 to enhance AMP expression in IECs. Acid metabolites from gut commensal fermentation of indigestible fibers, as well as local tissue acidification due to intestinal inflammation, may contribute to the activation of GPR65 signaling. Therefore, it seems reasonable to propose that activation of IEC-intrinsic GPR65 signaling in response to local acidification orchestrates optimal STAT3 phosphorylation downstream of IL-22 to optimize mucosal defensive responses. Previous studies have defined a dichotomous role of IEC-specific STAT3 in mediating resistance to DSS-induced epithelial damage, as well as in enhancing tumor growth.^52,53^ Hence, based on these bioinformatic findings, the impaired STAT3 downstream signaling and context-dependent role of STAT3 in different pathogenic milieu may account for the seemingly contradictory *in vivo* results.

As much of what has been learned about GPR65 function in IECs derived from studies based on mouse models and *in vitro* cell lines, we further examined the relevance to human IBD and potential translational value. We found a decrease in GPR65 expression in IECs from IBD patients and colitis mice, pointing to an important role for epithelial GPR65 in colitis development and progression. The decreased expression of GPR65 in inflamed epithelia seemed contradictory to our previous study which demonstrated enhanced expression of GPR65 in inflamed intestinal mucosa.^23^ It is generally accepted that GPR65 is mainly expressed in immune cells such as neutrophils, macrophages, NK cells and T lymphocytes according to previous studies, whereas the expression level in IECs is relatively low, which is consistent with our own work (data not shown). Considering the relatively high expression of GPR65 in immune cells but relatively low in IECs, it is reasonable to postulate that under inflammatory conditions, the decreased expression of GPR65 in inflamed epithelium was concealed by the augmented expression in inflammatory cells in lamina propria due to large inflammatory infiltrates and immune activation. Our current study indeed verified the expression of GPR65 in IECs and its functionality in IBD, further broadening the understanding of the role of epithelial GPR65.^22^ GPR65-mediated innate defense responses, including the production of AMPs in IECs, unequivocally clarify an attractive mechanism for therapeutic exploitation, notwithstanding some contextual discrepancies outlined above with respect to the capacity of GPR65 to affect the progression of colitis and CAC.

Given our finding that the mRNA and protein expression levels of epithelial GPR65 in IBD patients were significantly decreased, it can be suggested that the weakened GPR65 signaling might underlie increased susceptibility to IBD in these patients. As GPR65 is a susceptibility gene for human IBD, more efforts are needed to clarify the direct effects of the GPR65 variant on the functions of human IECs. Furthermore, comprehensive mechanistic insights concerning molecular biology and extrapolation of these findings to humans are still necessary to gain an in-depth understanding of the role of epithelial GPR65 in intestinal inflammation.

In summary, our findings provide essential insights into the immune mechanisms whereby GPR65 promotes host-microbial balance by regulating intestinal epithelial functions, and highlight the involvement of impaired epithelial GPR65 signaling in the etiology and pathology of IBD (Supplementary Figure S9). This study definitely provides the proof of concept that epithelial-derived GPR65 serves as a positive regulator and thus may be a potential therapeutic target to limit IBD initiation and development. Our current study has enriched the understanding of the field of host-microbial interactions, and future work will likely yield critical insights into this area and bring therapeutic innovation for IBD.

## Supplementary Material

Supplemental Material

## Data Availability

RNA sequencing data from this study are openly available in Gene Expression Omnibus at https://www.ncbi.nlm.nih.gov/bioproject (BioProject No. PRJNA967647). All other data supporting the findings of this study are available within the article and its supplementary materials or from the corresponding author upon reasonable request.

## Abbreviations

AMP: Antimicrobial peptide
CD: Crohn’s disease
*C. rodentium*: *Citrobacter rodentium*
CFUs: Colony-forming units
DEGs: Differentially expressed genes
DSS: Dextran sulfate sodium
GO: Gene Ontology
GSEA: Gene set enrichment analysis
GPCRs: G protein-coupled receptors
HC: Healthy controls
H&E: Hematoxylin and eosin
IBD: Inflammatory bowel disease
IECs: Intestinal epithelial cells
KEGG: Kyoto Encyclopedia of Genes and Genomes
PCA: Principal component analysis
PCoA: Principal coordination analysis
UC: Ulcerative colitis
