# Supplementary material for: Intestinal epithelial pH-sensing receptor GPR65 maintains mucosal homeostasis via regulating antimicrobial defense and restrains gut inflammation in inflammatory bowel disease
Source: Gut Microbes. 2023 Sep 25;15(2):2257269. doi: 10.1080/19490976.2023.2257269 (PMC10524779; doi:10.1080/19490976.2023.2257269)
Supplement: Supplemental Material [file KGMI_A_2257269_SM4370.zip › KGMI_SUPPLEMENTAL MATERIALS/Supplementary Figure legends.docx]

**Supplementary Figure Legends**

**Supplementary Figure 1. The generation and breeding roadmap of** ***Gpr65* conditional knockout mice and confirmation of IEC-specific depletion of GPR65.** (a) The breeding roadmap of *Gpr65* conditional knockout mice. (b) PCR validation of Gpr65 conditional knockout mice. (c) *Gpr65* mRNA expression in IECs isolated from the large bowel and small bowel of *Gpr65*^ΔIEC^ and *Gpr65*^fl/fl^ mice. (d) Surface GPR65 expression in EpCAM^+^CD45^-^ epithelial cells isolated from *Gpr65*^ΔIEC^ and *Gpr65*^fl/fl^ mice by flow cytometry. **p < 0.01, ****p < 0.0001. Data are representative of three independent experiments.

**Supplementary Figure 2. IEC-derived GPR65 does not impact on the development and differentiation of different specialized IEC lineages.** (a) H&E and Alcian blue staining of colon and ileum tissues from 8-week-old *Gpr65*^ΔIEC^ and *Gpr65*^fl/fl^ mice. Scale bars, 100 μm. Immunofluorescence staining of lysozyme in the distal ileum of *Gpr65*^ΔIEC^ and *Gpr65*^fl/fl^ mice. Scale bars, 100 μm. (b, c) qRT-PCR analysis of indicated genes from the colonic IECs of *Gpr65*^ΔIEC^ and *Gpr65*^fl/fl^ mice at the mRNA level. (d) qRT-PCR analysis of the indicated genes from colon tissues of *Gpr65*^ΔIEC^ and *Gpr65*^fl/fl^ mice at the mRNA level. Data are representative of three independent experiments.

**Supplementary Figure 3.** ***Gpr65*^ΔIEC^ mice show dysregulated fecal microbiota.** (a) Shannon-Wiener curve. (b) Rank-abundance distribution curve. (c) Chao index. (d) Simpson index. (e) LEfSe cladogram plot of the discriminative microbial taxa. The color represents the mouse group and the size of the circle represents the relative abundance of the taxa.

**Supplementary Figure 4.** ***Gpr65*^ΔIEC^ mice are subject to DSS-induced colitis.** (a, b) Increased numbers of CD4^+^ T cells, MPO^+^ neutrophils and F4/80^+^ macrophages in colon tissues from *Gpr65*^ΔIEC^ mice after DSS administration. Scale bars, 100 μm. **p < 0.01. Data are representative of three independent experiments.

**Supplementary Figure 5.** ***Gpr65*^ΔIEC^ mice are resistant to** **AOM/DSS-induced CAC.** (a) The body weight changes during the AOM/DSS-induced CAC. (n = 8-10 in each group) (b) Gross morphology of *Gpr65*^ΔIEC^ and *Gpr65*^fl/fl^ mice on day 76 of the CAC model. (c-e) Colons were opened longitudinally, and the tumor numbers and tumor size were measured. (f) The tumor size distribution according to tumor numbers was recorded. (g) H&E and immunohistochemical staining for Ki67 and PCNA in paraffin-embedded sections of adenoma-containing colons. Scale bars, 100 μm. *p < 0.05, **p < 0.01. Data are pooled from two independent experiments.

**Supplementary Figure 6.** ***Gpr65*^ΔIEC^ mice are susceptible to *C. rodentium*-induced colitis.** (a) General appearance of spleens of *Gpr65*^ΔIEC^ and *Gpr65*^fl/fl^ mice. (b, c) Immunohistochemical staining for CD4, MPO and F4/80 in colon tissues from *Gpr65*^ΔIEC^ and *Gpr65*^fl/fl^ mice. Original magnification, × 200. (d) Relative *Il22* mRNA expression in the distal colon tissues of indicated groups of mice. *p < 0.05, **p < 0.01 and ***p < 0.001. Data are representative of three independent experiments.

**Supplementary Figure 7. GPR65 deficiency induces significant transcriptome changes in mouse colonic IECs.** Colonic IECs from both *Gpr65*^ΔIEC^ and *Gpr65*^fl/fl^ mice were isolated for RNA sequencing after elimination of CD45^+^ immune cells. (a) The purity of colonic epithelial cells before and after magnetic bead sorting. (b) Heatmap of DEGs in colonic epithelial cells between *Gpr65*^ΔIEC^ and *Gpr65*^fl/fl^ mice. (c) Volcano plot showing upregulated and downregulated genes. (d) DEGs (fold change > 2.0 or < 0.5 and Padj < 0.01) between the two groups of mice were enriched for GO functional analysis by Metascape. Top 20 pathways from the indicated comparisons are shown. n = 3 biologically independent samples per group.

**Supplementary Figure 8. Pivotal signaling pathways related to antimicrobial defense in colonic IECs were regulated by GPR65.** (a, b) GSEA and heatmap plot showing downregulation of GO pathways “Positive regulation of immune response” and “Defense response to bacterium” in GPR65-deficient IECs. (c, d) GSEA and heatmap plot showing downregulation of KEGG pathways “Inflammatory bowel disease” and “JAK-STAT signaling pathway” in GPR65-deficient IECs. (e) Human colonic epithelial cell lines including SW480, HCT116, HIEC, Caco2 and HT29 were stimulated with or without rmIL-17A (100 ng/mL) and rmIL-22 (100 ng/mL) for 12 h, and relative mRNA expression of *REG3A* was evaluated by qRT-PCR. (f) Murine colon adenocarcinoma-derived epithelial cell line MC38 was stimulated with or without rmIL-17A (100 ng/mL) and rmIL-22 (100 ng/mL) for 12 h, and relative mRNA expression of *Reg3g* was evaluated by qRT-PCR. Data are representative of three independent experiments.

**Supplementary Figure 9. IEC-intrinsic GPR65 signaling maintains epithelial antimicrobial defense and protects from colitis.**

IEC-intrinsic GPR65 synergizes with IL-22-STAT3 signaling to drive optimal antimicrobial defense, and protects against gut microbiota dysbiosis and intestinal mucosal inflammation. Decreased level of GPR65 was found in IBD patients and damaged GPR65 signaling may confer an augmented susceptibility to intestinal inflammatory diseases.
