# Supplementary material for: Intestinal epithelial pH-sensing receptor GPR65 maintains mucosal homeostasis via regulating antimicrobial defense and restrains gut inflammation in inflammatory bowel disease
Source: Gut Microbes. 2023 Sep 25;15(2):2257269. doi: 10.1080/19490976.2023.2257269 (PMC10524779; doi:10.1080/19490976.2023.2257269)
Supplement: Supplemental Material [file KGMI_A_2257269_SM4370.zip › KGMI_SUPPLEMENTAL MATERIALS/Supplementary Methods.docx]

**Supplementary Methods**

**Immunohistochemical staining**

Freshly isolated colon was fixed in 10% formalin and embedded in a paraffin block followed by cutting into 5 μm slices. These sections were then transferred onto glass slides for further use. After deparaffinization, rehydration and antigen retrieval, these slides were treated with 0.3% Triton X-100 for 10 min at room temperature and blocked with 10% normal donkey serum for 1 h. Subsequently, immunostaining was performed with rabbit anti-mouse CD4 (ab183685, Abcam), rat anti-mouse F4/80 (ab100790, Abcam), rabbit anti-mouse MPO (ab208670, Abcam), rabbit anti-mouse Ki67 (ab15580, Abcam) or rabbit-anti mouse PCNA (ab92552, Abcam) overnight at 4°C. The slips were then treated with 3% H_2_O_2_ for elimination of endogenous peroxidase. After 3 washes with PBS, the slides were incubated with horseradish peroxidase-conjugated goat anti-rabbit/rat secondary antibodies for 30 min. The color reaction was developed with 3,3’-diaminobenzidine, and the slides were counterstained with hematoxylin. Images were observed under optical microscopy.

**Transcriptomic data analysis**

Raw data (raw reads) of fastq format were firstly processed through in-house perl scripts. In this step, clean data (clean reads) were obtained by removing reads containing adapter, reads containing ploy-N and low-quality reads from raw data. At the same time, Q20, Q30 and GC content the clean data were calculated. All downstream analyses were based on the clean data with high quality. Reference genome and gene model annotation files were downloaded from genome website directly. Index of the reference genome was built using Hisat2 v2.0.5 and paired-end clean reads were aligned to the reference genome using Hisat2 v2.0.5. FeatureCounts v1.5.0-p3 was used to count the reads numbers mapped to each gene. FPKM (fragments per kilobase of exon per million reads/fragments mapped) of each gene was then calculated based on the length of the gene and reads count mapped to this gene. Differential expression analysis was performed using the DESeq2 R package (1.16.1). Genes with an adjusted p-value < 0.05 found by DESeq2 were assigned as differentially expressed. For Gene Ontology (GO) and KEGG enrichment analysis, differentially expressed genes were implemented by the clusterProfiler (<https://bioconductor.org/packages/release/bioc/html/clusterProfiler.html>) and Metascape. Heatmaps were generated using the pHeatmap R package (<https://CRAN.R-project.org/package=pheatmap>).

**Isolation of intestinal crypts and organoid culture assay**

The intestinal crypts were isolated and cultured according to manufacturer’s instructions (06010, StemCell; Vancouver, BC, Canada). Briefly, colons were isolated, opened longitudinally and flushed thoroughly. The colon was cut into 2 mm pieces and the rinsing procedure was repeated about 15 times with cold PBS. The tissue pieces were then resuspended in Gentle Cell Dissociation Reagent and incubated at room temperature (15 - 25°C) for 20 minutes on a rocking platform at 20 rpm. The villous materials and tissue fragments were discarded and the crypts were collected after centrifugation at 290×g for 5 min at 4°C. The pelleted intestinal crypts were resuspended with 300 μL mixture organoid medium (complete IntestiCult^™^ Organoid Growth Medium: Matrigel^®^ Matrix = 1:1) and seeded in a pre-warmed 24-well plate. The plate was incubated at 37°C for 10 minutes to set the Matrigel, and then overlaid with 750 μL complete organoid medium. The complete organoid medium was changed every 2 days and the organoids were passaged every 10 days. The crypt growth and morphology were monitored under bright field microscope.^1^

**Intestinal permeability assay**

Mice were starved but with free water overnight before gavaged with 600 mg/kg FITC-dextran (FD4; Sigma-Aldrich) in a total volume of 200 μL. After 4 hours, mice were sacrificed and fluorescence intensity of FITC-dextran in sera was measured by Cytation 5 Cell Imaging Multimode Reader (BioTek) with an excitation of 485 nm and an emission wavelength of 528 nm.

**Gene set enrichment analysis (GSEA)**

GSEA was performed based on the GO Biological Processes, Kyoto Encyclopedia of Genes and Genomes database and Hallmark gene sets with the “clusterProfiler” package of R.^2^ A false discovery rate (FDR) less than 0.25 and an absolute value of the normalized enrichment score (NES) greater than 1 were defined as the cutoff criteria.

**AOM/DSS-induced CAC mouse model**

AOM/DSS-induced CAC mouse model was conducted as described previously.^3^ Briefly, *Gpr65*^ΔIEC^ mice and *Gpr65*^fl/fl^ littermates were intraperitoneally injected with AOM (12 mg/kg; Sigma-Aldrich). 7 days later, mice were administered with 1.5% DSS in the drinking water for 7 consecutive days, followed by 14 days of regular water for recovery. This cycle was repeated three times, and all mice were sacrificed for analysis on day 76 of the experiments.

**References**

1. Pleguezuelos-Manzano C, Puschhof J, van den Brink S, Geurts V, Beumer J, Clevers H. Establishment and Culture of Human Intestinal Organoids Derived from Adult Stem Cells. Current protocols in immunology. 2020;130(1):e106. doi:10.1002/cpim.106.

2. Wu T, Hu E, Xu S, Chen M, Guo P, Dai Z, et al. clusterProfiler 4.0: A universal enrichment tool for interpreting omics data. Innovation (Cambridge (Mass)). 2021;2(3):100141. doi:10.1016/j.xinn.2021.100141.

3. He Q, Gao H, Chang YL, Wu X, Lin R, Li G, et al. ETS-1 facilitates Th1 cell-mediated mucosal inflammation in inflammatory bowel diseases through upregulating CIRBP. Journal of autoimmunity. 2022;132102872. doi:10.1016/j.jaut.2022.102872.
