# Supplementary material for: Intestinal epithelial pH-sensing receptor GPR65 maintains mucosal homeostasis via regulating antimicrobial defense and restrains gut inflammation in inflammatory bowel disease
Source: Gut Microbes. 2023 Sep 25;15(2):2257269. doi: 10.1080/19490976.2023.2257269 (PMC10524779; doi:10.1080/19490976.2023.2257269)
Supplement: Supplemental Material [file KGMI_A_2257269_SM4370.zip › KGMI_SUPPLEMENTAL MATERIALS/Supplementary Tables.docx]

**Supplementary Tables**

**Supplementary Table 1.** The list of Primers used for qRT-PCR analysis

| Gene | Species | Forward Sequence (5’ to 3’) | Reverse Sequence (5’ to 3’) |
| --- | --- | --- | --- |
| *GAPDH* | Human | GGAGCGAGATCCCTCCAAAAT | GGCTGTTGTCATACTTCTCATGG |
| *REG3A* | Human | TATGGCTCCCACTGCTATGCCT | TCTTCACCAGGGAGGACACGAA |
| *GPR65* | Human | TGATCTGCAACCGGAAAGTCT | TCAGCAACATCACATGAAAGGG |
| *Gapdh* | Mouse | GGTTGTCTCCTGCGACTTCA | TGGTCCAGGGTTTCTTACTCC |
| *Reg3g* | Mouse | ATGCTTCCCCGTATAACCATCA | GGCCATATCTGCATCATACCAG |
| *Reg3b* | Mouse | ACTCCCTGAAGAATATACCCTCC | CGCTATTGAGCACAGATACGAG |
| *Nos2* | Mouse | TTGGGTCTTGTTCACTCCACGG | CCTCTTTCAGGTCACTTTGGTAGG |
| *Gpr65* | Mouse | TTCAGTCTGCCTGCATCAGT | CGGGAGGGGTATTTAGTCCTT |
| *Muc2* | Mouse | ATGCCCACCTCCTCAAAGAC | GTAGTTTCCGTTGGAACAGTGAA |
| *Lyz1* | Mouse | GTCACACTTCCTCGCTTTCC | TGACTGTCACCAGCATCCAT |
| *Dclk1* | Mouse | TGAAGCGCCTGTACACTCTG | CTTCTCTGGTCCACATGCAA |
| *Lgr5* | Mouse | CAGCCTCAAAGTGCTTATGCT | GTGGCACGTAACTGATGTGG |
| *Defb1* | Mouse | AGGTGTTGGCATTCTCACAAG | GCTTATCTGGTTTACAGGTTCCC |
| *Defb3* | Mouse | AAAGGAGGCAGATGCTGGAA | ACGGGATCTTGGTCTTCTCT |
| *Defb4* | Mouse | GCAGCCTTTACCCAAATTATC | ACAATTGCCAATCTGTCGAA |
| *Zo1* | Mouse | GCTTTAGCGAACAGAAGGAGC | TTCATTTTTCCGAGACTTCACCA |
| *Claudin1* | Mouse | GGGGACAACATCGTGACCG | AGGAGTCGAAGACTTTGCACT |
| *Claudin2* | Mouse | CAACTGGTGGGCTACATCCTA | CCCTTGGAAAAGCCAACCG |
| *Claudin5* | Mouse | GCAAGGTGTATGAATCTGTGCT | GTCAAGGTAACAAAGAGTGCCA |
| *Occludin* | Mouse | TTGAAAGTCCACCTCCTTACAGA | CCGGATAAAAAGAGTACGCTGG |
| *Il22* | Mouse | ATGAGTTTTTCCCTTATGGGGAC | GCTGGAAGTTGGACACCTCAA |
| *Il22bp* | Mouse | TCAGCAGCAAAGACAGAAGAAAC | GTGTCTCCAGCCCAACTCTCA |
| *Il6* | Mouse | TAGTCCTTCCTACCCCAATTTCC | TTGGTCCTTAGCCACTCCTTC |
| *Tnfa* | Mouse | CATCTTCTCAAAATTCGAGTGACAA | TGGGAGTAGACAAGGTACAACCC |
| *Cxcl1* | Mouse | CTGGGATTCACCTCAAGAACATC | CAGGGTCAAGGCAAGCCTC |
| *Lcn2* | Mouse | TGGCCCTGAGTGTCATGTG | CTCTTGTAGCTCATAGATGGTGC |

**Supplementary Table 2.** Primers of bacteria used for qRT-PCR analysis

| Species | Forward Sequence (5’ to 3’) | Reverse Sequence (5’ to 3’) |
| --- | --- | --- |
| *C. rodentium* | GCTTCTGCGAAGTCTGTCAA | CAGTAAAGCGACTTAACAGATT |
| *16S rRNA* | 515F: GTGCCAGCAGCCGCGGTAA | 806R: GGACTACCAGGGTATCTAAT |
