# Supplementary figures and images for: Intestinal epithelial pH-sensing receptor GPR65 maintains mucosal homeostasis via regulating antimicrobial defense and restrains gut inflammation in inflammatory bowel disease
Source: Gut Microbes. 2023 Sep 25;15(2):2257269. doi: 10.1080/19490976.2023.2257269 (PMC10524779; doi:10.1080/19490976.2023.2257269)

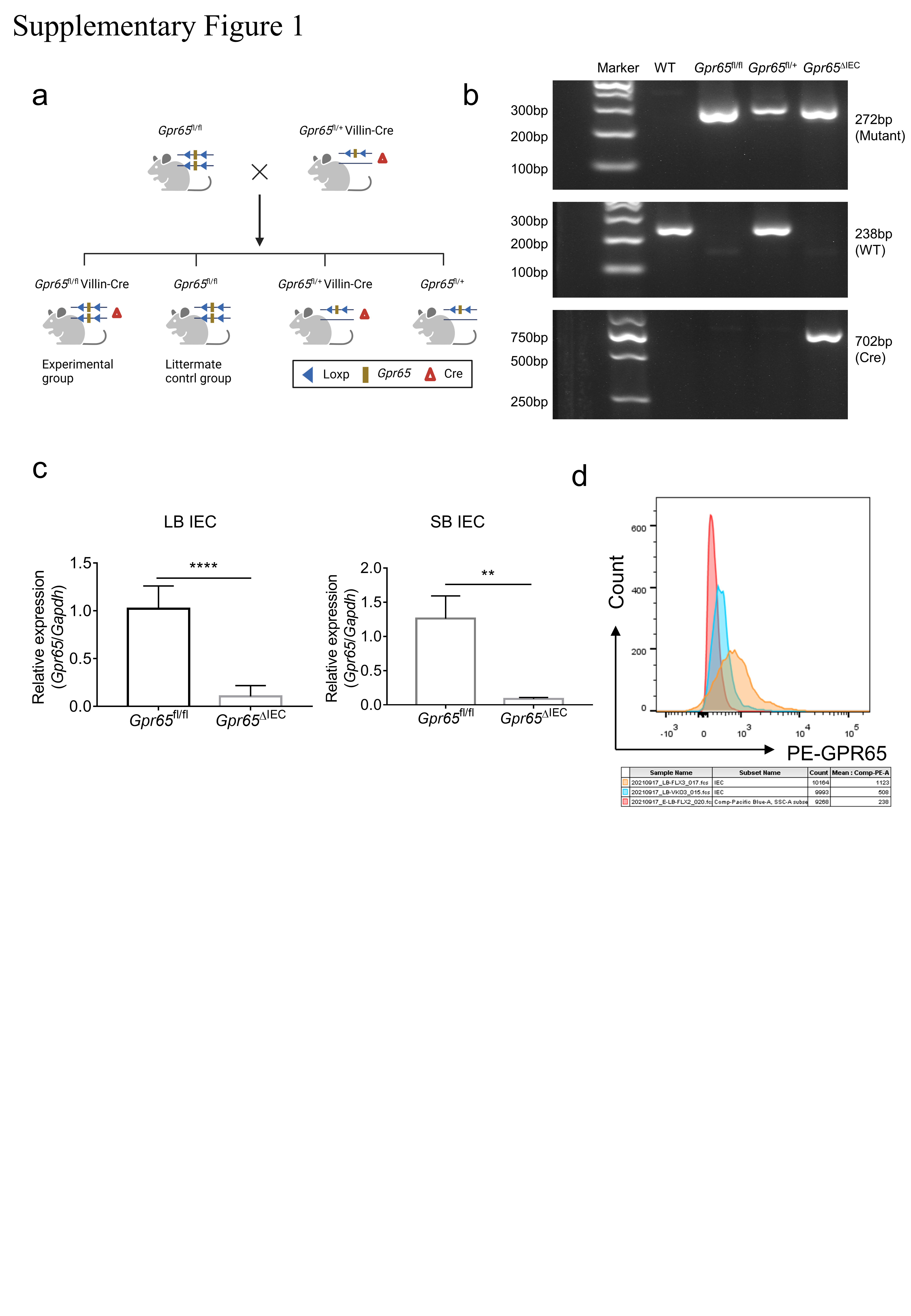

Supplement: Supplemental Material [file KGMI_A_2257269_SM4370.zip › KGMI_SUPPLEMENTAL MATERIALS/Supplementary Figure 1.TIF]

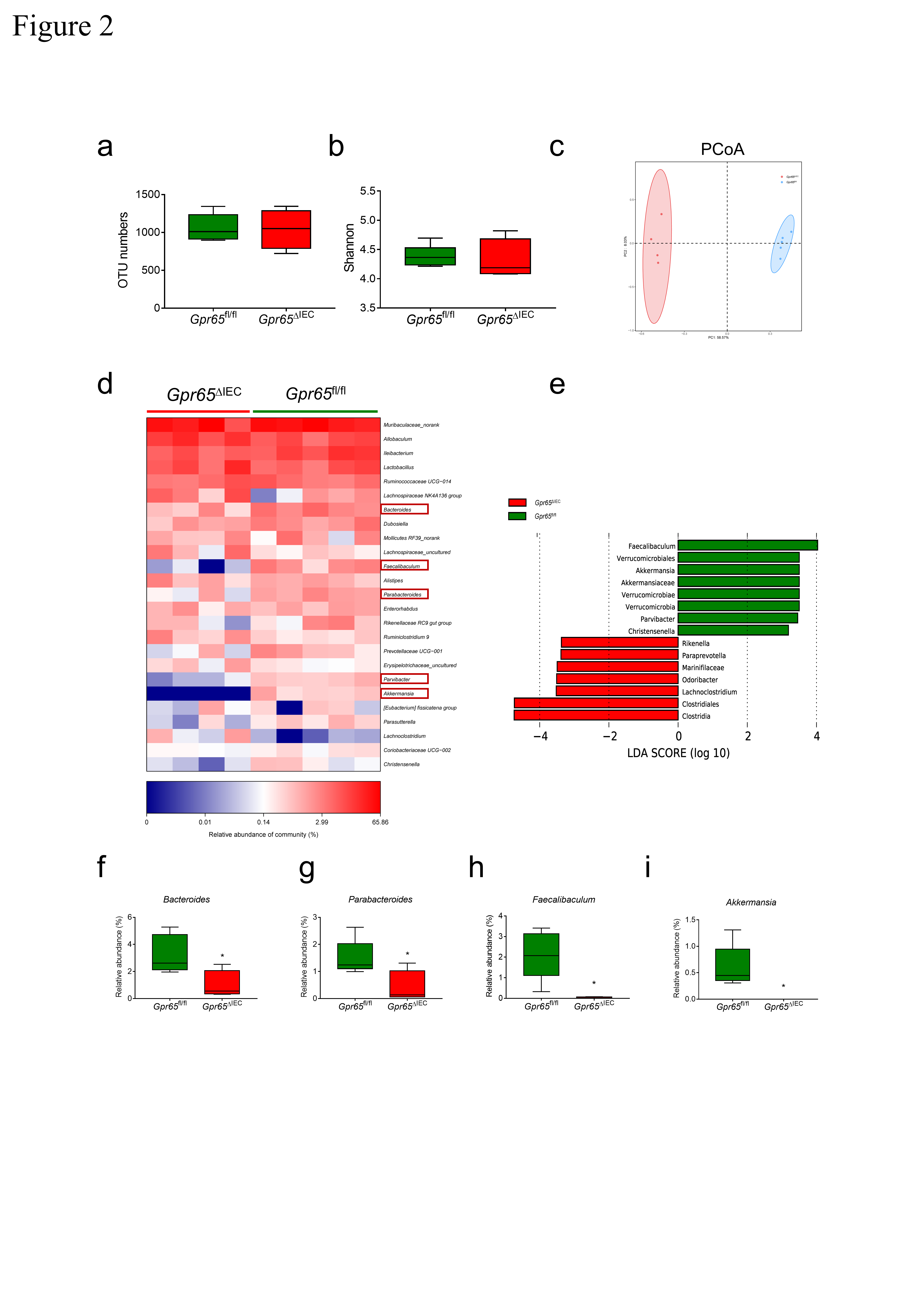

Supplement: Supplemental Material [file KGMI_A_2257269_SM4370.zip › KGMI_SUPPLEMENTAL MATERIALS/Supplementary Figure 2.TIF]

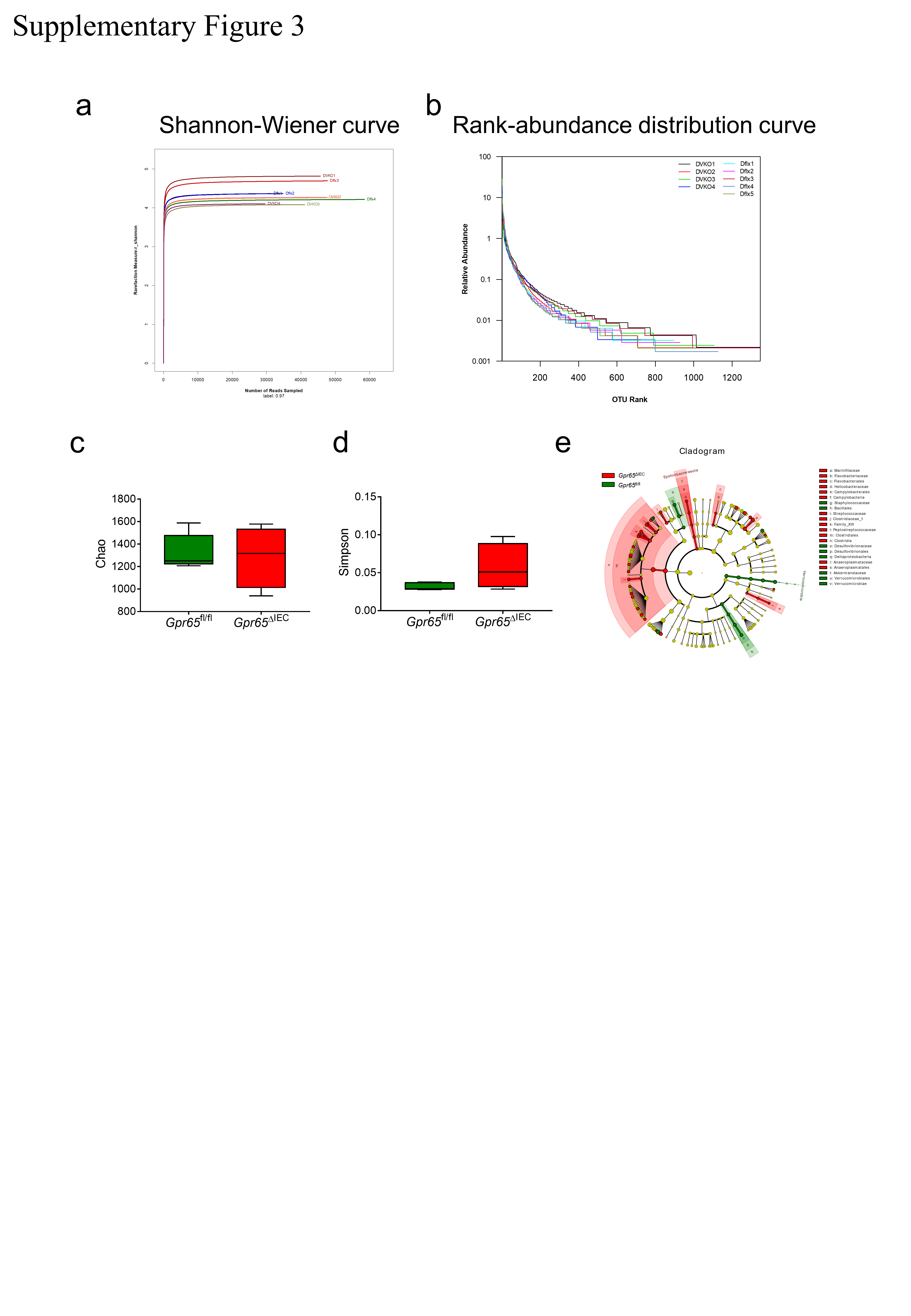

Supplement: Supplemental Material [file KGMI_A_2257269_SM4370.zip › KGMI_SUPPLEMENTAL MATERIALS/Supplementary Figure 3.TIF]

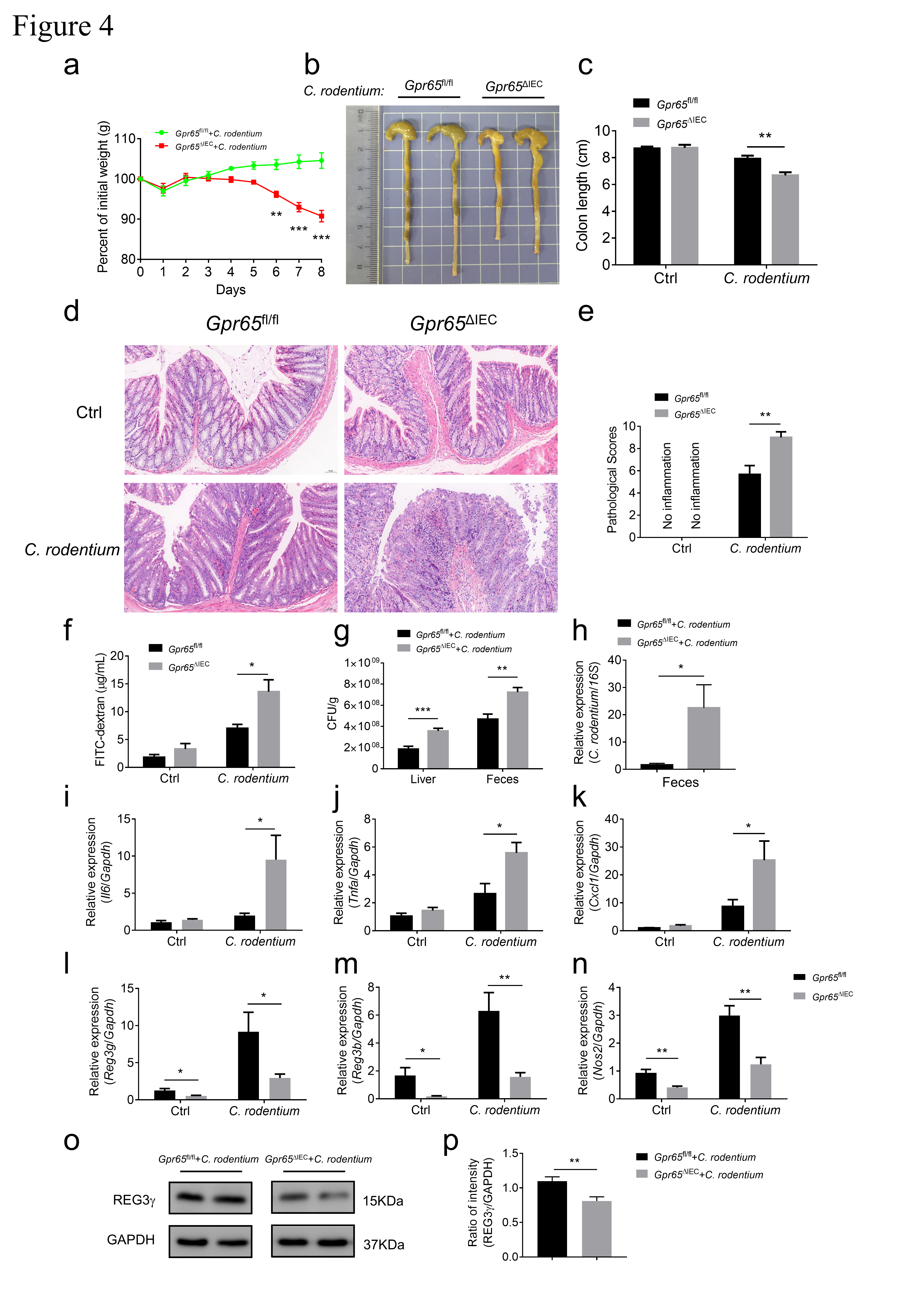

Supplement: Supplemental Material [file KGMI_A_2257269_SM4370.zip › KGMI_SUPPLEMENTAL MATERIALS/Supplementary Figure 4.TIF]

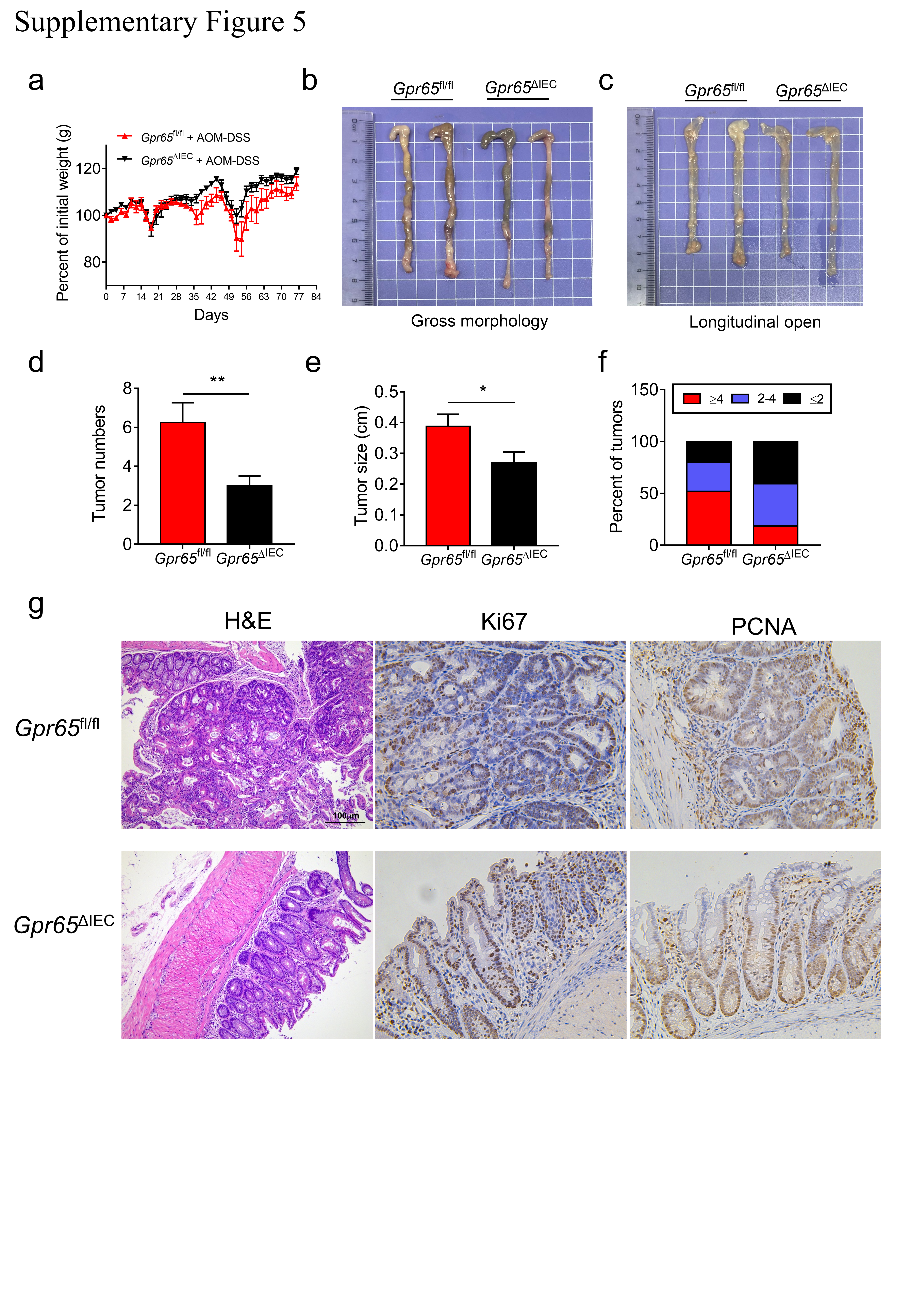

Supplement: Supplemental Material [file KGMI_A_2257269_SM4370.zip › KGMI_SUPPLEMENTAL MATERIALS/Supplementary Figure 5.TIF]

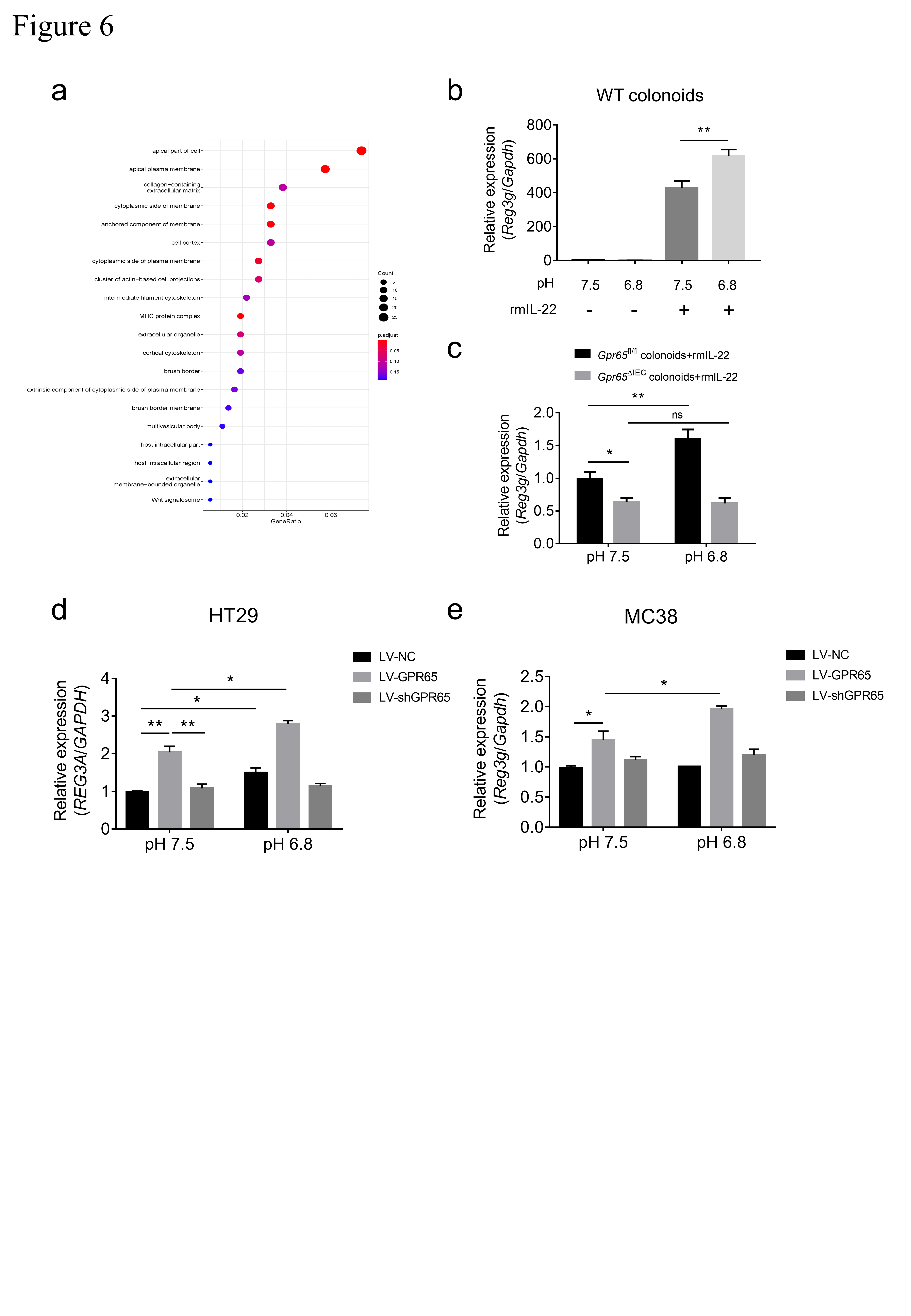

Supplement: Supplemental Material [file KGMI_A_2257269_SM4370.zip › KGMI_SUPPLEMENTAL MATERIALS/Supplementary Figure 6.TIF]

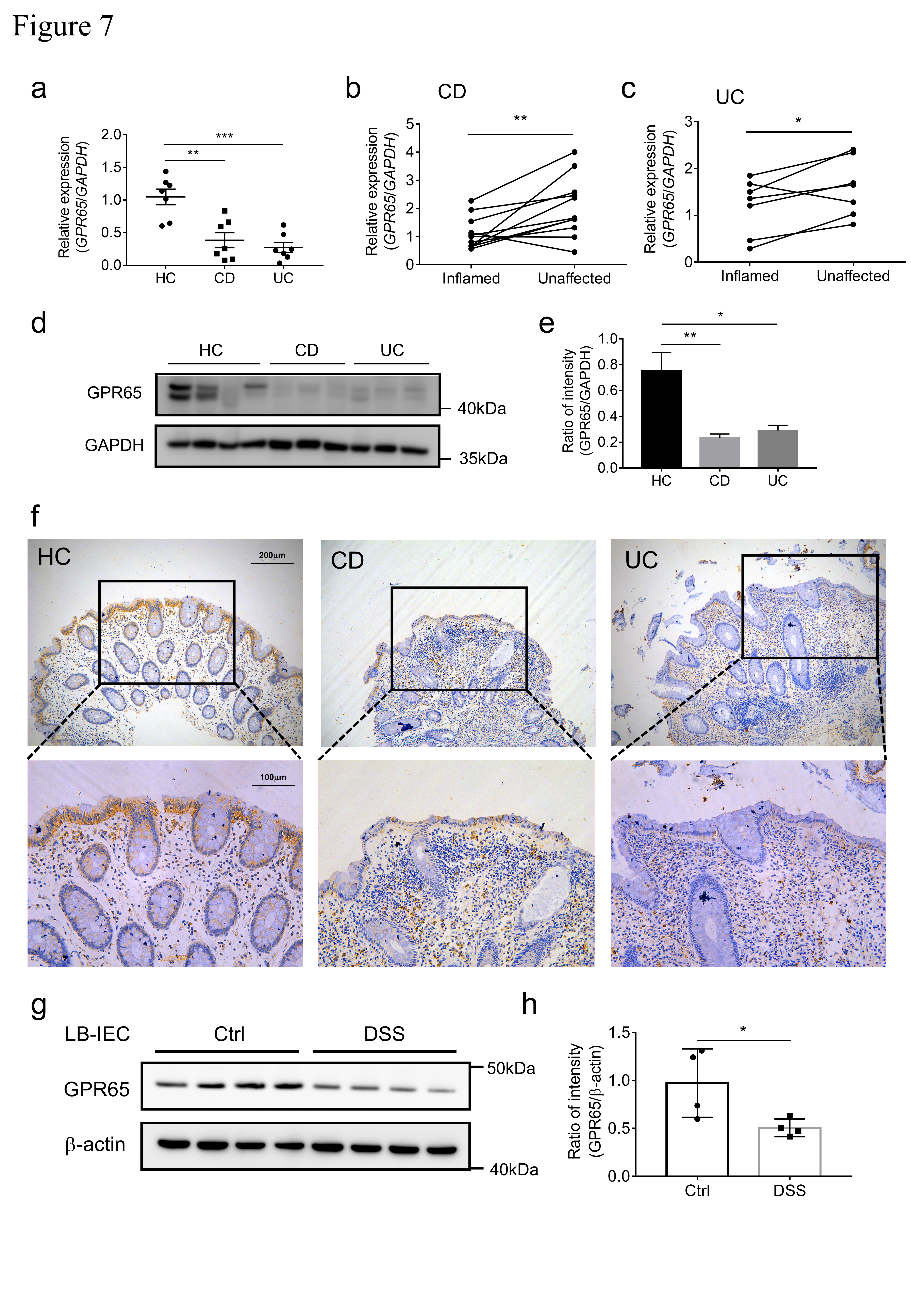

Supplement: Supplemental Material [file KGMI_A_2257269_SM4370.zip › KGMI_SUPPLEMENTAL MATERIALS/Supplementary Figure 7.TIF]

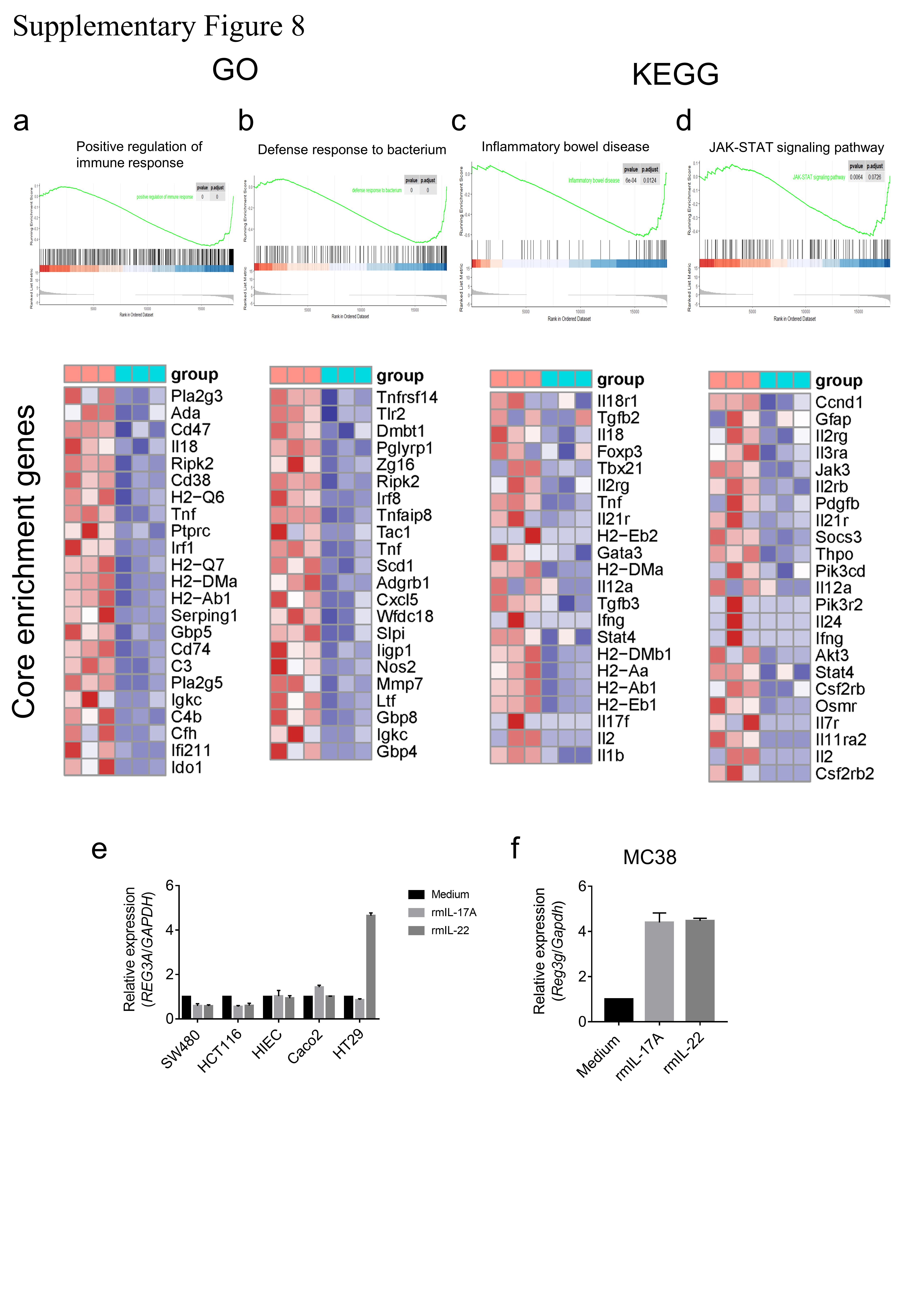

Supplement: Supplemental Material [file KGMI_A_2257269_SM4370.zip › KGMI_SUPPLEMENTAL MATERIALS/Supplementary Figure 8.TIF]

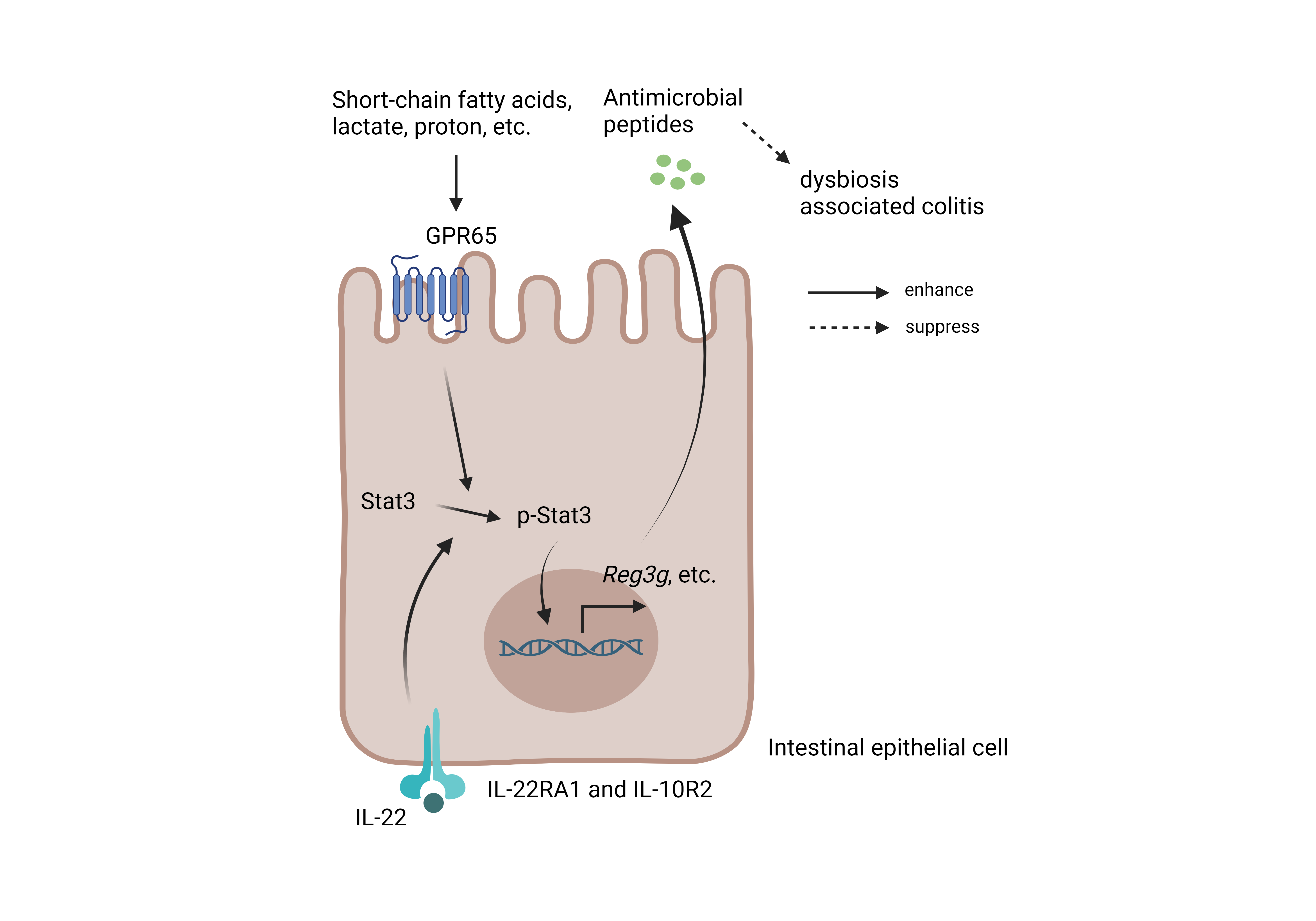

Supplement: Supplemental Material [file KGMI_A_2257269_SM4370.zip › KGMI_SUPPLEMENTAL MATERIALS/Supplementary Figure 9.png]
